# Synaptic vesicle recycling: steps and principles

**DOI:** 10.1002/embj.201386357

**Published:** 2014-03-04

**Authors:** Silvio O Rizzoli

**Affiliations:** Department of Neuro- and Sensory Physiology, University Medical Center Göttingen, European Neuroscience InstituteGöttingen, Germany

**Keywords:** clathrin, endocytosis, exocytosis, SNAREs, vesicle pools

## Abstract

Synaptic vesicle recycling is one of the best-studied cellular pathways. Many of the proteins involved are known, and their interactions are becoming increasingly clear. However, as for many other pathways, it is still difficult to understand synaptic vesicle recycling as a whole. While it is generally possible to point out how synaptic reactions take place, it is not always easy to understand what triggers or controls them. Also, it is often difficult to understand how the availability of the reaction partners is controlled: how the reaction partners manage to find each other in the right place, at the right time. I present here an overview of synaptic vesicle recycling, discussing the mechanisms that trigger different reactions, and those that ensure the availability of reaction partners. A central argument is that synaptic vesicles bind soluble cofactor proteins, with low affinity, and thus control their availability in the synapse, forming a buffer for cofactor proteins. The availability of cofactor proteins, in turn, regulates the different synaptic reactions. Similar mechanisms, in which one of the reaction partners buffers another, may apply to many other processes, from the biogenesis to the degradation of the synaptic vesicle.

## Introduction

Chemical synapses release neurotransmitter from small, round, seemingly identical organelles – the synaptic vesicles (SVs). These fuse with the plasma membrane and release their contents of neurotransmitter molecules (exocytosis). The molecules diffuse across the gap between the pre- and postsynaptic neuronal membranes leading to the activation or inhibition of the postsynaptic compartment. The SV components are subsequently retrieved from the plasma membrane of the presynaptic neuron (endocytosis) and are turned into a new fusion-competent SV. The best name for this process has been proposed by Heuser and Reese (1973): *synaptic vesicle recycling* (see also reviews in Sudhof, 2004; Haucke *et al*, 2011). This term underlines one of the major characteristics of the exo- and endocytosis process: it goes on throughout the lifetime of the organism, creating and re-creating the SVs hundreds or thousands of times. This process must go on without significant mistakes, as these would lead to lethal consequences by impairing neuronal communication.

It is therefore not surprising that neuroscientists see SV recycling as one of the best-controlled processes in cell biology. However, it is still unclear how recycling is controlled. Many of the effector molecules are known, but what controls SV recycling as a whole? What ensures the presence of the many exo- and endocyosis cofactors in the synapse? How do the cofactors find their targets at the right time? What controls the number of vesicles that are exo- or endocytosed, preventing the synapse membrane from expanding to an impossible size or from eating itself up through excessive retrieval?

Perhaps specific proteins control these processes. However, this answer is insufficient, since it stops short of asking what ensures the presence of the control proteins in the right places, and what triggers their activity at the right time (Saka & Rizzoli, 2012). An alternative answer is that each of the cellular reactions is controlled by the concentrations of the reaction partners, rather than by special control proteins. The positions, numbers and reactive states of the different partners regulate the way individual reactions go, and thus ultimately control the overall activity of the synapse.

A critical point is that most reactions are localized. For example, all synaptic reactions are localized to the synaptic bouton, which brings up the question of how the various soluble proteins are maintained there. It has been suggested that the cluster of SVs within the synapse binds to and buffers a plethora of soluble binding partners of SVs, including proteins involved in exo- and endocytosis (Shupliakov, 2009; Denker *et al*, 2011a,2011b). In this fashion the SVs may provide a mechanism to control the abundance of their partners in the synapse, and ultimately to control exo- and endocytosis reactions. This type of reaction, in which one partner is present in high numbers, and buffers the other partner(s), may be involved in numerous reactions of the synaptic vesicle pathway. Importantly, the less mobile reaction partner is always the one to buffer its mobile counterpart.

**Figure 1:**
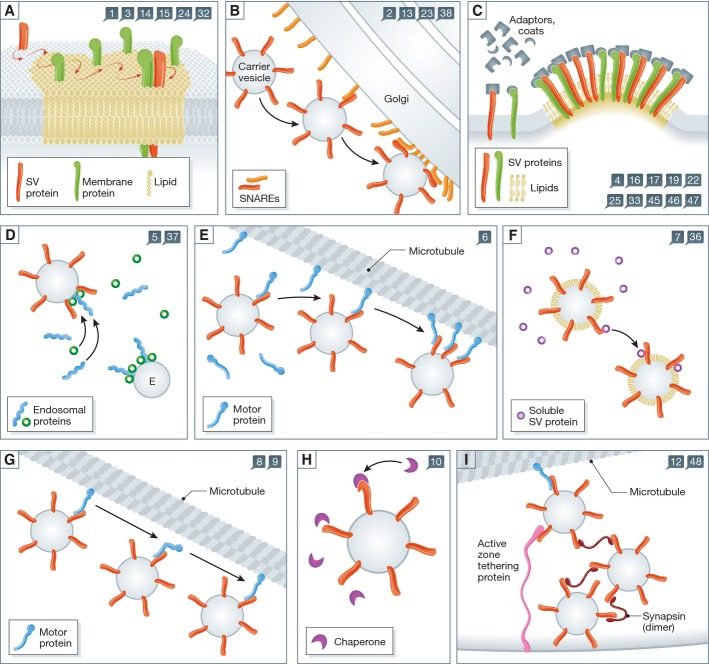
Different types of reactions that occur during vesicle recycling The numbers in the blue squares indicate the reaction steps addressed by the schemes. (A) A SV protein has entered an organelle and is sorting in the membrane. It encounters a domain of lipids and proteins that it has a limited affinity for, and diffuses slowly within the domain. It eventually becomes stabilized within the domain by binding multiple partners (lipids, proteins). (B) A carrier vesicle fuses with an organelle, such as the Golgi apparatus. The vesicle is buffered locally by the surface of the Golgi apparatus, which is enriched in molecules involved in docking and tethering carrier vesicles, as well as in the fusion with these vesicles (SNARE proteins). The meeting of fusion molecules from the two membranes triggers the fusion of the organelles. (C) Budding reactions. The accumulation of several types of SV molecules in a membrane domain triggers the recruitment of several types of adaptor and coat proteins, each binding to its own preferred SV partner. The accumulation of the coats and adaptors eventually surpasses a critical mass and thus induces the budding reaction. (D) Processing through an endosome. Components of the endosomal pathway, such as Rabs and their effectors, are recruited to carrier vesicles, by interacting with multiple components (proteins, lipids) of the vesicles. This gives the carrier vesicles and endosomal nature, and allows them to fuse to endosomes. Note that this is a putative step in SV recycling. (E) The carrier vesicle interacts with a motor protein, whose high affinity for microtubules causes the eventual delivery of the carrier to the microtubule bundle. Here it may bind further motors, and may proceed along the microtubule toward the synapse. (F) Soluble proteins are recruited onto the carrier vesicle, by interacting with its protein and lipid components. (G) Progression of the vesicle along microtubules (anterograde transport). (H) Recruitment of chaperone proteins onto the carrier vesicle. The chaperone is buffered by the vesicle through low-affinity interactions with normal proteins, until binding more strongly to a spontaneously unfolded protein. In this way the chaperones “probe” continually the surface of the vesicle, and can rapidly and efficiently detect unfolded elements. (I) The carrier vesicle comes off the microtubule track, by interacting with docking proteins and/or with other “sticky” proteins such as synapsin. These multiple interactions are stronger than the interaction with the motor, and remove the vesicle from the microtubule.

**Figure 2:**
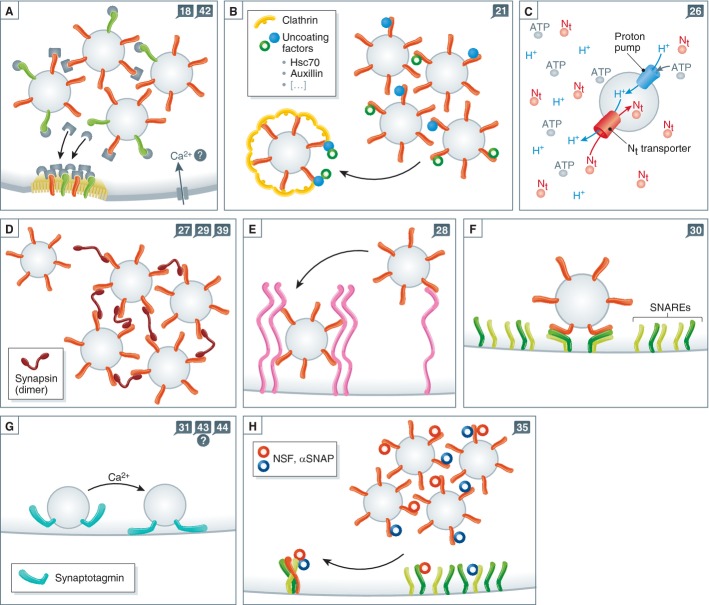
More types of reaction schemes from vesicle recycling As in Fig 1, the numbers in the blue squares indicate the reaction steps addressed by the schemes. (A) In order for endocytosis to happen, adaptor and coat proteins need to be recruited from a source within the synapse. This source may be the SV cluster: many adaptors and coat proteins may be bound onto SV proteins at rest. They may be released during activity and will participate in endocytosis. (B) Similar to panel A, uncoating factors may be recruited from the vesicle cluster onto a coated vesicle. (C) The reactions involved in neurotransmitter refilling: vATPase molecules acidify the SV, and neurotransmitter molecules enter it. (D) The SV may become entangled in a synapsin meshwork, by spontaneous binding to one or more synapsin molecules. (E) Docking at the active zone – in the same fashion as in panel D, but through interactions with active zone proteins. (F) The SV engages plasma membrane SNAREs and prepares (in a sense) for fusion. The reaction is relatively similar to the one from panels D-E, with SNAREs being the interacting molecules. (G) Calcium stimulates fusion by interactions with sensor proteins such as synaptotagmin. (H) As in panels A or B, α-SNAP and NSF are recruited onto SNARE complexes either from the vesicle cluster or from SNAREs on the plasma membrane. The result is the separation of SNARE complexes.

**Figure 3:**
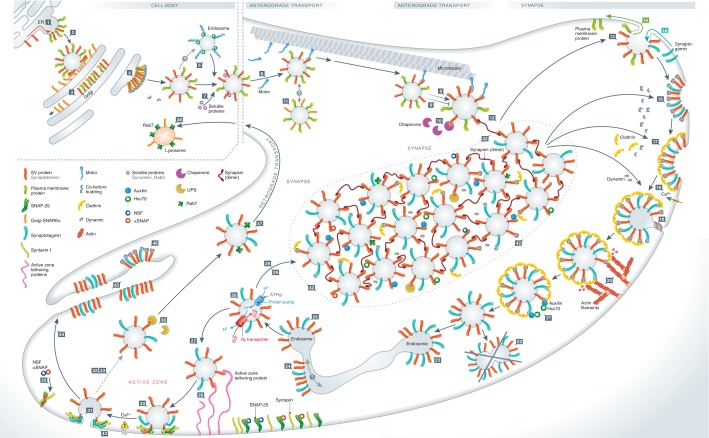
Overview of synaptic vesicle recycling New SV proteins are generated in the ER and diffuse to specific domains (step 1), before budding and fusion of the carrier vesicle to the Golgi apparatus (step 2). Sorting in the Golgi apparatus, in which some contaminant molecules are removed (orange; step 3), is followed by budding from the Golgi apparatus (step 4). The new carrier vesicle may sort through an endosome (step 5), and will interact with motor proteins to reach microtubules (step 6). Soluble SV proteins may bind specific components of the carrier and be transported along (step 7). Other proteins may also tag along, such as chaperones (step 10), through non-specific interactions with the carrier vesicle proteins. Anterograde transport follows (step 8); it will be blocked if any damage to microtubules takes place (step 9). It is doubtful whether there is any fusion between carrier vesicles along the way (step 11). The carrier eventually comes off microtubules in the synapse (step 12), and will fuse to the plasma membrane (step 13). Sorting of contaminants follows (green, step 14), in parallel with recruitment of other SV proteins (blue, step 15). Budding from the plasma membrane follows (steps 16, 17, 18 and 19), and the coated vesicle is pushed by actin away from the membrane (step 20), before uncoating (step 21). The newly uncoated vesicles do not fuse to each other (step 22), but may fuse to an endosome (step 23), which is followed by endosomal sorting (step 24) and budding (step 25). The new SV fills with neurotransmitter (step 26). The SV either remains mobile for a while (steps 27, 29), docks at the active zone (step 28), or becomes integrated in the SV cluster (step 39). Priming for fusion (step 30) follows docking, and in turn is followed by fusion, upon action potential stimulation and calcium entry (step 31) or in spontaneous fashion, independent of stimulation (step 43). Sorting of SV components may happen in the plasma membrane, to be eventually followed by endocytosis (steps 32, 33). Before endocytosis the SNARE complexes that formed during fusion are separated (step 35), which is an important sorting step for SV components. Damaged SV proteins may be targeted by the proteasomal system (step 36; note that this reaction is likely to happen almost exclusively for soluble SV proteins, although for simplicity the SV protein is depicted here on the SV membrane). Damaged SVs may be tagged for retrograde transport (step 37). Lysosomal degradation awaits (step 38). The SV cluster forms a reserve for the various soluble proteins involved in SV recycling (step 42). Finally, strong synaptic stimulation results in massive exocytosis (step 44) and formation of membrane infoldings (step 45), from which endocytosis machinery removes SV-sized chunks (step 46).

To understand the problem of regulating (controlling) synaptic reactions in more depth, I present below an overview of synaptic vesicle recycling, step by step, in which I repeatedly ask the question of how the reactions are triggered, and how they may be controlled (see Figs1 and 2 for different reaction types, and Fig 3 for an overview of the synaptic vesicle cycle).

However, there is a tendency to see cellular reactions as linear, textbook style schemes, organized by “control proteins”. I have therefore also included here two boxes dealing with simple, common sense considerations on how such reactions should be regarded, if one is to consider the complexity of the cellular environment. Box 1 presents the basic set of principles that separates a cellular reaction from a text book scheme. Box 2 speculates on how such reactions could be triggered and controlled in a simple fashion.

**Box 1: A set of principles for cellular and synaptic reactions**

**Figure:**
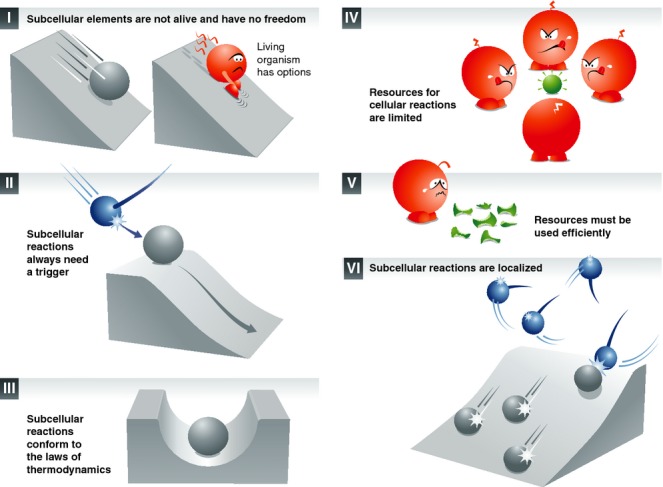

Textbook knowledge indicates that the life of the cell is maintained by different organelles and proteins which have different functions. These functions are complementary, and all organelles and proteins thus work together for the good of the cell.

This view is almost entirely wrong. It is based on descriptions of cells as building blocks of tissues and organisms, written especially during the 19th century (see for example Virchow, 1862). Cells with different functions were suggested to collaborate for the good of the organism (Virchow, 1862). This may be true at the cellular level, but it is not so at the sub-cellular level.

Organelles and proteins do not have functions. While there are many definitions of the word “function”, they all concur on the fact that the function is the “thing” (activity, etc.) that something or someone does, or is intended to do, or is employed to do, or is particularly fitted to do. Organelles and proteins do not “do” anything, and are not intended to “do” anything. They only participate in particular reactions, should the conditions surrounding them allow for the particular reactions. The opposite view, in which the organelles and proteins “do” something, is nonsensical in philosophical terms, since it endows these sub-cellular elements with life (see for example one of the earliest and simplest definitions of life, in Plato's *Phaedrus* 245e; Plato, 1997). To provide a simple comparison for this, nobody would claim that the function of a key is to open a lock, or even that *the key opens the lock*. The act of opening the lock only takes place when the conditions surrounding the key are favorable: when a hand introduces the key into the lock and turns. The hand, not the key, opens the lock.

Saying that organelles and proteins have functions is convenient, since it removes the need to ask how they are organized and controlled. Saying that *the key's function is to open the lock* removes any need to wonder how it finds itself in front of the lock. The hand does not need to be looked for. In other words, the semantic implications of the word “function” make us ignore the fact that a hand is needed to first position and then turn the key.

However, if we embrace the (self-evident) view that organelles and proteins have no functions, we are faced with a collection of organelles and proteins that somehow participate in reactions that maintain the life of the cell. One now needs to wonder how the different reactions can be achieved: how the different elements (organelles, proteins) find themselves in the right places, at the right times, and how the reaction may be initiated. For this, I formulate here a few common-sense principles that help in setting the framework.

- *There is no life below the cell level*. Sub-cellular elements, including proteins, organelles or synapses, are not alive. They are subject to the direct influence of the (cellular) environment, and have no choice or freedom in their reactions. The freedom to choose a course of action (a “function”) is an essential attribute of life (Rousseau, 1914) – not of the reactions that a non-living, sub-cellular element can take part in.
- *There is always a reaction trigger*. The reactions in which sub-cellular elements are involved are initiated by a cause in the (cellular) environment.
- *Non-living entities tend to lose energy*. Sub-cellular reactions conform to the laws of thermodynamics. Each sub-cellular reaction involves a degree of energy loss (through friction, heat production, etc.), which is compensated for by the energy intake of the living cell.
- *Resources are limited*. Both energy resources (ATP, metabolites) and reaction partners for sub-cellular reactions (cofactors) are present in finite quantities, which may be limiting for the various reactions. The time in which cellular reactions need to be finished is also limited.
- *Resources must be used efficiently*. A living cell must minimize the energy expended. The number of steps in a reaction must be minimized. The quantity of sub-cellular elements (proteins, organelles) necessary for the reaction must be kept to a minimum.
- *Most sub-cellular processes are localized. S*ub-cellular reactions take place at defined locations, such as synapses, specific organelles, membrane domains or protein clusters.

**Box 2: Possible control mechanisms in a cellular reaction**

Using the principles noted in Box 1, I focus on a cellular reaction involving two sub-cellular elements (proteins, organelles, etc.), A and B, which react and form the product AB only when the cell requires it. I discuss how the localization of the elements may be controlled, and how the reaction may be initialized. The reaction might be very simple: A and B interact spontaneously and form the product AB, just as two chemicals may do in a test tube. However, this situation must be exceedingly rare in the cell: for an efficient cellular reaction to take place, both A and B need to find each other not only at the right location but also at the right time: they need to form AB only at the right time, neither too late, nor too soon. This observation is especially relevant for the synapse: for any reaction to take place, the two partners must first locate to the synapse, which is unlikely to happen spontaneously, since all the elements of the synapse are produced far away in the cell body.

In a textbook situation, A and B would simply “know” their function – but this cannot be the case. So, how do A and B find each other? Let us start by assuming that A is the less mobile of the two elements. A will move within the cell until it reaches a location where the energy it receives from the environment is not sufficient to cause its dispersal (to induce it to diffuse away). This situation can be easily imagined for a cholesterol-binding protein that encounters a cholesterol patch on the plasma membrane, or for a membrane adhesion protein that encounters an extracellular binding partner. One key aspect of this situation is that the entry of A into the location is accompanied by energy loss. The greater this loss, the more stably will A be bound within the location.

B, the more mobile element, may or may not use the same pathway to reach the location. It often cannot, especially when A is attached to a membrane and B is cytosolic. How does B reach, nevertheless, the location? One possibility is to have B in ample concentrations throughout the entire volume of the cell. This would contradict the efficiency principle (Box 1, v) twice: first, much larger quantities of B are produced than are actually needed. Second, A and B would interact continually, instead of producing the time-controlled reaction needed by the cell. Another possibility is to generate an independent control system for the mobile reaction partner – which, however, would also contradict the efficiency principle, by increasing the number of steps required by the reaction. A third alternative is offered by the binding affinity between A and B: this will impact on the localization of B, restricting its diffusion to areas around A.

**Figure:**
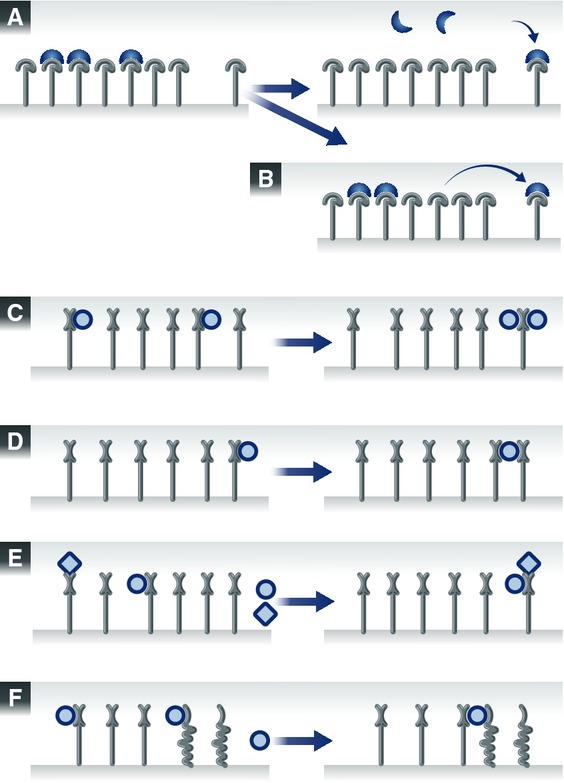

The problem with this alternative is that a single copy of A is not sufficient to localize B. The solution would be to produce more copies of A. They would all reach the same area, since they have the same energetic requirements, and would form a buffer for the B molecules in that particular location. At cell level, the energy loss due to the increased production of A is amply compensated for by the much lower production of B.

A and B are thus both present at the location, although A is there in larger numbers than absolutely necessary to produce the amounts of AB the cell needs. At the same time, AB products would form continually. The answer to this problem is to have most A copies in a less than fully reactive state, A’. Examples include proteins that self-assemble in tight clusters (as for the SNARE fusion protein syntaxin 1; Sieber *et al*, 2007) or the SVs, the majority of whom are kept immobile and fusion-incompetent by cross-linking molecules such as synapsin (Hirokawa *et al*, 1989). A’ molecules can still bind B, but cannot proceed to the formation of AB: a non-productive interaction A'B takes place, possibly with lower affinity. The buffering of B still takes place, but AB is not constantly produced, since only a few “lucky” A molecules are in reaction-competent state at any one time.

Note, however, that the buffering effect increases the number of B molecules at the expense of their mobility, with the buffered B molecules finding themselves in A'B complexes. The concentration of *free* B molecules does not increase due to buffering effects. Nevertheless, the system already meets almost all the conditions listed above: A and B are at the location, and the reaction does not take place, except for a few spontaneous AB complexes that form between non-buffered B and non-reserve A (as in the spontaneous complexing of SNARE molecules or spontaneous vesicle fusion Sudhof, 2004; Denker & Rizzoli, 2010). To set A and B in “motion,” a cause external to A and B is needed: a motion trigger. This external cause induces the bufferedB molecules to form numerous AB complexes with the non-reserve A molecules. The cause can act in several ways (see Fig 1A–F):

- It liberates B from the buffer (e.g., stimulation and calcium liberate mobile cofactor proteins from synaptic vesicle clusters Shupliakov, 2009; Denker *et al*, 2011)
- It increases the affinity of the non-reserve A for B, above the affinity that B has for the A’ molecules (for example, some SV proteins are recognized by endocytosis cofactors with much higher affinity in the plasma membrane, after exocytosis, than on the non-exocytosed vesicles; see step 16).
- The cause may also represent the spontaneous meeting of two B molecules with one A molecule. The resulting product is ABB. The affinity of the A+B+B interaction is much higher than that of A+B interactions. A’ molecules are here A molecules that have only met one B molecule (or none). These last two considerations apply to the following reactions as well (d–f).
- Conversely, the cause may be the spontaneous meeting of two A molecules with one B molecule, producing AAB.
- As a variant of reaction (c), the two B molecules may be different (B_1_ and B_2_). Both are buffered by A, but neither AB_1_, nor AB_2_ are productive interactions. The reaction needed by the cell requires all three components, resulting in AB_1_B_2_. The cause is the spontaneous interaction between A, B_1_ and B_2_.
- Similar to (d): the reaction between two different A molecules (A_1_ and A_2_) and B, resulting in A_1_A_2_B.

The first two reactions (a, b) are tightly controlled temporally. The following four (c–f) will take place spontaneously, and the reaction frequency will be controlled by the concentrations and locations of the reaction partners. All these reactions result in reasonably time-controlled cellular events that take place in an efficient fashion. They probably represent a basic level of control – the basic level at which each reaction in, for example, synaptic vesicle recycling is regulated.

## From protein biogenesis to the synapse

The first question to be answered is how the SV protein gets out of the ER, how it progresses to the next compartment, the Golgi apparatus, and eventually to the synapse (since little protein translation occurs in the axon (Taylor *et al*, 2013). This is one of the least understood aspects of synaptic biology.

It is possible that each SV protein is targeted by specialized machinery for delivery to the synapse. This, however, would imply an excessive effort for the neuron, in generating and maintaining the different targeting machineries. Alternatively, a few important SV proteins may be targeted by specialized machineries (detailed below), while other SV proteins just tag along and are co-transported by sharing the same domains on membranes, or the same carrier organelles. This may be the case especially for proteins that are highly abundant in the secretory pathway, such as the SNAREs syntaxin 1 and SNAP-25 (Takamori *et al*, 2006; Sieber *et al*, 2007; Knowles *et al*, 2010).

One proposal is that a subset of SV proteins and lipids spontaneously form a proto-SV domain, on the ER and/or on the Golgi membrane, in which other SV proteins are co-sorted. A protein such as the abundant synaptophysin (Takamori *et al*, 2006; Mutch *et al*, 2011) may associate with lipids such as cholesterol (Thiele *et al*, 2000) and possibly with other proteins such as synaptobrevin 2 (VAMP2), to form the proto-SV domain. This domain later controls the sorting of proteins into or out of the nascent vesicle (Pennuto *et al*, 2003). This would explain the role of synaptophysin in SV endocytosis (Kwon & Chapman, 2011), and especially in the sorting of synaptobrevin (Gordon *et al*, 2011). It has even been suggested that synaptophysin expression induces the formation of small vesicles in cells that do not normally form such organelles (Leube *et al*, 1989, 1994), although this is a highly contested view (Johnston *et al*, 1989; Cameron *et al*, 1991; Linstedt A & Kelly, 1991; Régnier-Vigouroux *et al*, 1991). Finally, a related protein, synaptogyrin, also seems to be involved in the same process of quality control of vesicle biogenesis or endocytosis, and thus may be partially redundant with synaptophysin (Stevens *et al*, 2012).

A problem with this proposal is that knocking out synaptophysin or synaptogyrin (Stevens *et al*, 2012) results in minor phenotypes (see below, step 15). However, knock-out experiments only indicate how an organism deals with the lack of a protein, not what reactions the protein may be involved in. For example, *Drosophila* flies lacking the main calcium sensor of stimulated synaptic activity, synaptotagmin I, are amazingly fit, and even reach adulthood (Loewen *et al*, 2001).

### 1 Formation of a new SV protein and its diffusion to a specific domain in the ER (Fig 1A)

**Table:** 

Reaction	Diffusion of a transmembrane SV protein to a particular membrane domain in the ER, enriched in SV proteins.
Partners	SV protein (mobile), and ER domain containing SV proteins and/or lipids (less mobile).
The trigger	Liberation of SV protein from ribosome machinery.
The control mechanism	The proteins/lipids in the ER domain interact with and buffer the SV protein, causing it to be enriched in the particular domain.

A transmembrane protein is translated in the rough endoplasmic reticulum (ER) in the neuronal cell body. The protein belongs to one of the following three categories: proteins enriched in SVs (termed “SV proteins” throughout the rest of this review), proteins that are present in most membranes of the secretory pathway, or proteins that avoid the SVs (see Takamori *et al*, 2006 for the categorization of SV proteins).

It is likely that the newly produced protein first diffuses freely into the ER membrane, where it interacts with different proteins and lipids. Low-affinity interactions delay its diffusion when the protein reaches membrane domains containing, for example, synaptophysin, cholesterol or specific phospholipids known to interact with SV proteins (Thiele *et al*, 2000; Van den Bogaart *et al*, 2011; Khuong *et al*, 2013) or to be important in SV recycling (Dason *et al*, 2010). In such domains the SV protein has a much higher chance of interacting with multiple SV-specific binding partners (cofactors) than elsewhere, resulting in its recruitment here (see for example the interactions between the different subunits of the vacuolar proton-ATPase, vATPase (Stevens & Forgac, 1997); or the potential interactions of SV2 and synaptotagmin (Yao *et al*, 2010).

Some proteins may have a more complex fate, before ending in an ER domain. A special case is the abundant SV fusion protein synaptobrevin 2, a member of the SNARE family (also termed VAMP2; see Jahn & Scheller, 2006 and step 11 for a description of SNARE fusion proteins). This tail-anchored protein is first translated in the cytosol and then interacts with members of the GET (guided entry of tail-anchored proteins) pathway, to have its hydrophobic C-terminus post-translationally inserted into the ER membrane (Kutay *et al*, 1995).

The SV protein or protein domain will eventually find itself in an endoplasmic reticulum export site (ERES), either by random diffusion or by interacting with elements stabilized in these areas, for example coat proteins involved in ER-to-Golgi transport (COPII). This will lead to the inclusion of the protein in carrier vesicles or tubules forming at the ERES.

### 2 ER-to-Golgi traffic (Fig 1B)

**Table:** 

Reaction	Fusion of an ER carrier vesicle to the Golgi apparatus.
Partners	Carrier vesicle (mobile), and domain on the surface of the Golgi apparatus that contains docking/tethering factors and SNARE proteins (virtually immobile, by comparison with the carrier vesicle).
The trigger	Unclear. Possibly the spontaneous assembly of SNARE proteins from carrier vesicle and Golgi apparatus, after docking has been achieved through the interaction of docking/tethering molecules.
The control mechanism	The presence of numerous docking/tethering proteins, as well as SNAREs, on the surface of the Golgi causes repeated interactions of the carrier vesicle with this surface (i.e., buffer the vesicle to this surface). Fusion eventually takes place when sufficient SNAREs meet to form complexes.

The newly formed vesicles or tubules will then fuse to the Golgi apparatus. The traffic of SV components between the ER and the Golgi is likely non-specific, with these proteins following the general flow of membrane traffic between the two organelles (Watson & Stephens, 2005). The table indicates the putative fusion reaction between the ER carrier vesicle and the Golgi apparatus: the vesicle explores the surface of the *cis*-Golgi by low-affinity interactions, until a suitable site is found, where fusion proteins (SNAREs, step 11) can engage with high affinity and induce the collapse of the two membranes.

### 3 Sorting in the Golgi apparatus

Reaction table similar to that for step 1.

Just as in the ER, the proteins diffuse to proteolipid patches, and may form partially stable protein/lipid assemblies (as described for the plasma membrane (Simons & Ikonen, 1997)). The interactions between the different SV proteins established in the ER, such as the synaptobrevin/synaptophysin heterodimers (Siddiqui *et al*, 2007), will likely persist in the Golgi membrane. As indicated in the introduction to the biogenesis section, cholesterol may stabilize such a proto-SV domain (Bennett *et al*, 1992; Jia *et al*, 2006). This is likely especially in view of the large amount of cholesterol contained by mature SVs (Takamori *et al*, 2006).

### 4 Budding from the Golgi apparatus (Fig 1C)

**Table:** 

Reaction	Budding of a precursor vesicle from the Golgi apparatus.
Partners	Soluble budding cofactors (including coat proteins and their adaptors), and SV proteins and/or lipids arranged in domains on the Golgi surface (less mobile than the soluble proteins).
The trigger	Unclear. Possibly the accumulation of soluble cofactors onto the SV protein/lipid domain, beyond a critical mass. This induces a chain reaction in which more cofactors bind and eventually pinch off the vesicle.
The control mechanism	The amount of SV proteins and/or lipids in the domain on the Golgi surface controls the binding of cofactors to this area. Subsequently, the cofactor-bound surface determines the binding of further cofactors, eventually completing the reaction.

The formation of vesicles or vesicle precursors from the Golgi apparatus and/or the endosomal system is not well understood. These steps are difficult to study, from a technical point of view, and the results are also difficult to interpret. Budding is likely to proceed through the involvement of different adaptors and coats, recruited individually by the different SV proteins or assemblies of SV proteins (Popoff *et al*, 2011). Different machineries may target individual SV proteins (Hannah *et al*, 1999), based on signals that direct their inclusion in the SVs. It is unclear whether signals for the removal of proteins from nascent vesicles are necessary (Hannah *et al*, 1999; Prado & Prado, 2002). The interactions of the SV proteins with endocytosis or endosomal proteins in the Golgi apparatus are based on various sorting signals (Grote *et al*, 1995; West A *et al*, 1997), including both classical dileucine-based signals (Santos *et al*, 2009) or more specialized sorting motifs (Koo *et al*, 2011), whose discussion, however, is beyond the purpose of this review.

One major question is whether the Golgi or endosomal system will produce SVs or only precursor vesicles, which require additional sorting in the synapse to become SVs. Vesicles or tubules of varying sizes and shapes appear to be produced (Tsukita & Ishikawa, 1980; Nakata *et al*, 1998), depending upon their composition and the resulting differential recruitment of adaptors and coats. SV-sized organelles may also be produced: for example, small SV-like vesicles accumulate in the cell bodies of neurons that lack a motor protein involved in axonal transport (Yonekawa *et al*, 1998). Thus, it is difficult to answer this question based on morphology alone. The framework presented above provides a possible interpretation. A SV requires at least five types of major transmembrane proteins: (i) the SNARE synaptobrevin, for fusion; (ii) synaptotagmin, for calcium detection; (iii) the neurotransmitter transporter, to fill the vesicle with transmitter molecules; (iv) SV2, probably involved in release (Wan *et al*, 2010); (v) the poorly understood, but immensely abundant synaptophysin (Takamori *et al*, 2006). The cell produces the proteins in an uncoordinated (or poorly coordinated) fashion. Therefore, the proteins do not find themselves in the Golgi in the right stoichiometric proportions for the formation of complete, perfect SVs: one protein or another will always be rather scarce at any one point in time. This implies that the Golgi will be unable to form perfect SVs, and will only produce SV precursors.

At the same time, the synaptic vesicle proteins will interact with different endocytosis or endosomal proteins (coat proteins, adaptors, cofactors). Single SV molecules cannot attract sufficient cofactors, but the multiple molecules stabilized in a patch of membrane (step 3) will be sufficient to act as a buffer and recruit the soluble coat molecules. The newly recruited coat molecules buffer additional cofactors, and probably also additional SV proteins that may still be mobile in the Golgi membrane. Eventually a critical mass of cofactors and SV proteins is attained that induces the full budding reaction (see table). This reaction is similar to that of endocytosis of SV proteins from the plasma membrane of the synapse (McNiven & Thompson, 2006; Popoff *et al*, 2011), but not identical: in the Golgi the emerging vesicle will not contain all the SV proteins, and thus not all the cofactors recruited by the full complement of a SV can be recruited to the incomplete SV patch in the Golgi.

### 5 Post-Golgi endosomal processing? (Fig 1D)

**Table:** 

Reaction	Formation of a domain of endosomal proteins on the surface of the precursor vesicle, which later allows this vesicle to dock to and fuse to a sorting endosome.
Partners	Soluble docking/tethering cofactors, including endosomal Rab molecules (mobile) and the carrier vesicle (much less mobile).
The trigger	Unclear. As at step 4, possibly the accumulation of soluble cofactors onto an unknown protein/lipid domain on the surface of the precursor vesicle.
The control mechanism	Unclear. A similar mechanism to step 4 could be proposed, in which some unknown factors from the surface of the precursor vesicle buffer locally endosomal Rab proteins and/or other membrane organizing molecules (Zerial & McBride, 2001; Stenmark, 2009).

Scission from the Golgi membrane places the SV proteins in a new environment, a new organelle: the vesicle precursor. Does this precursor vesicle fuse now with the endosomal system, for further sorting? The process is not sufficiently known to accurately answer this question. In neuroendocrine cells most of the SV proteins can be found in endosomes (Donnert *et al*, 2006, 2007), although it is unclear whether the proteins have reached the endosomes directly from the Golgi or after endocytosis from the plasma membrane (see below steps 23–25). Of course, endosomal sorting could help eliminate contaminants from the precursor vesicle. However, it is unclear why this would require fusion to the endosomal system: would not sorting and budding from the TGN or the precursor organelle suffice?

### 6 Finding motors for anterograde transport (Fig 1E)

**Table:** 

Reaction	Recruitment of the precursor vesicle onto microtubules, for anterograde transport.
Partners	First, motor proteins in soluble form interact with the less mobile precursor vesicle. Second, the precursor vesicle, decorated by motor proteins, is buffered onto the immobile microtubules, by interactions mediated by the motor proteins.
The trigger	Unclear. When do the motors bind the precursor vesicles? How long does it last until they reach the microtubules? Do motor proteins bind the SV proteins already on the Golgi or ER membranes?
The control mechanism	First, the surface of the vesicle acts as a buffer system for the motor proteins, recruiting them locally. Second, the microtubule acts as a buffer system for the vesicle-associated motors. When and how the reactions start is unclear.

The precursor vesicles, which have a rather heterogeneous composition (due to the stoichiometry issue discussed above, step 4), need to be transported along the axon. The interaction of any one of the proteins found on the precursor vesicles with the right motor proteins will target the vesicles for axonal transport. Since not all SV proteins share the same precursors, they will not share the same motors either (Okada *et al*, 1995; Kaether *et al*, 2000). Interactions of SV proteins with motors include the recognition of precursor vesicles by the molecule DENN/MADD that binds simultaneously motor proteins and the SV protein Rab3 (Niwa *et al*, 2008), or interactions with the scaffolding protein liprin-α (Shin *et al*, 2003). For an in-depth discussion of motors and cargo recognition mechanisms see Hirokawa *et al,* 2010.

Some studies have indicated that SV proteins are co-transported, in packets of vesicles, together with other components such as active zone proteins (Ahmari *et al*, 2000; Wu *et al*, 2013). These proteins may inhabit approximately 80-nm dense-core vesicles (Zhai *et al*, 2001; Waites *et al*, 2005), which probably represent the basis for the formation of active zones. Despite frequent debates whether the SV and active zone precursors are delivered together, the association of their transport vesicles should not be surprising: many SV proteins have a tendency to bind active zone components, and this tendency can lead to no other result than the clustering of their respective vesicles. The two types of cargoes (SV proteins, active zone proteins) do not appear to mix within the same vesicles (Maas *et al*, 2012).

### 7 Finding motors for soluble proteins (Fig 1F)

**Table:** 

Reaction	Recruitment of soluble SV proteins onto the surface of the precursor vesicles.
Partners	Soluble SV proteins in cytosol (mobile) versus precursor vesicle (far less mobile).
The trigger	Spontaneous interaction of soluble proteins with precursor vesicle proteins and/or lipids.
The control mechanism	The surface of the vesicle acts as a buffer system for the soluble proteins, recruiting them locally. Multiple interactions with SV proteins and/or lipids may stabilize recruitment, to allow long-distance transport.

An interesting problem is the transport of soluble SV proteins along the axon. The early experiments of Baitinger and Willard (1987) demonstrated that a small fraction of a soluble protein, synapsin, moves as fast as the transmembrane SV components. However, the transport of the bulk of synapsin is much slower. Imaging experiments demonstrated 20 years later that the slow progress of soluble molecules is due to their moving “rapidly but infrequently, with pauses during transit” (Roy *et al*, 2007), in contrast to synaptophysin, which moved rapidly and continuously. The soluble proteins moved along microtubules (Roy *et al*, 2008; Scott *et al*, 2011), just as the transmembrane ones.

The following scheme for the transport of soluble SV proteins can be imagined: after translation they diffuse in the cytosol until they bind molecules that bring them to a low-energy state. Most of these proteins bind to SVs when found within the synapse (Takamori *et al*, 2006; Denker *et al*, 2011). A simple hypothesis is therefore that soluble proteins bind to SV precursors and are co-transported towards synapses. If the binding is very strong they will be transported about as fast as SV transmembrane proteins. If less strongly bound, most soluble proteins will come unbound during transport, and will therefore have to wait until the next vesicle precursor comes by.

An interesting alternative for soluble proteins that do not bind to SV proteins is that they may be transported along with the chaperone molecule heat-shock-cognate 70 (Hsc70), which can interact in a non-specific fashion with multiple proteins. Hsc70 is able to bind the transport machinery directly (Terada *et al*, 2010) and would thus ensure the co-transport of a variety of soluble cargoes. How cargo selection would be ensured by this mechanism is unclear.

### 8 Anterograde transport (Fig 1G)

**Table:** 

Reaction	Movement of the precursor vesicle along microtubules.
Partners	Precursor vesicle, decorated with motor proteins (mobile), and the microtubule (immobile).
The trigger	ATP hydrolysis by the motor.
The control mechanism	When the motor molecule unbinds from the microtubule, a possible result would be the diffusion of the precursor vesicle away from the microtubule. Is that prevented by the presence of multiple motor molecules on one vesicle, one of which must always be bound to the microtubule? Presumably pauses caused by accidental vesicle release may occur (Wu *et al*, 2013), but will be soon followed by renewed binding to microtubule.

The step that follows is the transport of the SV precursor towards the release sites. It will take place as long as the system receives ATP, and the microtubules are not interrupted. Motors such as KIF1A or KIF1Bβ transport the precursor packages (Vale, 2003) (see Nishinari *et al*, 2005 for a description of the transport mechanism). As for many other motors, the reaction includes the binding of the motor molecules onto the microtubule, followed by ATP hydrolysis and movement along the microtubule.

One important issue is that the cargo should not be lost: it should not remain “stuck” in the axon, and it should not be picked up by retrograde motors (such as dynein 1 or KIFC2 (Hirokawa *et al*, 2010)). We can hypothesize that SV protein doesn't bind both types of motors, since otherwise directed transport would be rather difficult to achieve. And, importantly, the protein that binds anterograde motors cannot be present on retrograde cargo, since otherwise this cargo could never leave the synapses. Rab3, one of the proteins that do interact with anterograde motors, albeit indirectly (see step 6) should therefore be degraded in the synapse, rather than sent for degradation in retrograde cargo.

What about soluble cargo? Some proteins will get unstuck and will have to wait for the next precursor (step 7). But some may prove more troublesome: several synaptic proteins have an inherent tendency to bind curved membranes – especially BAR-domain proteins such as endophilin and amphiphysin (Mim & Unger, 2012). They get recruited by curved membranes, irrespective of the biological significance of the curves, as demonstrated by poking and curving the membrane with metal nanocones (Galic *et al*, 2012). Thus, although these proteins are vital for synaptic vesicle recycling, they would get stuck on bends in the axonal membrane and would not reach the synapse, unless special mechanisms or structures keep the axon relatively smooth along its length. This may be ensured by circular actin structures lining the axon (Xu *et al*, 2013), which would limit the loss of curvature-binding proteins during transport.

### 9 Hitting a stop

Reaction table similar to that for step 8.

As long as the microtubules or the axons are not interrupted, transport continues towards the synapses. However, the transport route will become “clogged” upon nerve damage, and material will come off the microtubules and will accumulate on both sides of the damaged area. The material proximal to a nerve/microtubule damage site consists mainly of vesicle precursors and mitochondria (Li *et al*, 1992; Li & Dahlström, 1997).

### 10 Stability of proteins during transport (Fig 1H)

**Table:** 

Reaction	Damaged protein on the precursor vesicle meets chaperone, which helps in its refolding.
Partners	Protein on the precursor vesicle (less mobile) and the soluble chaperone (highly mobile).
The trigger	Spontaneous meeting of chaperone with damaged protein.
The control mechanism	Chaperones could not find rapidly the unfolded proteins on the precursor vesicle if they (chaperones) are randomly distributed at all times. It is more likely that the chaperones are buffered onto the surface of the vesicle precursors by repeated interactions with normal (not unfolded) proteins, which ensures the presence of the chaperones close to the proteins that may become unfolded.

An interesting question is how the long-term transport of the different proteins impacts their stability. The fast transport of vesicles reaches speeds of between 5 and 40 centimeters per day; the slower movement of soluble proteins averages only about 0.8 cm per day (Hirokawa *et al*, 2010). Some of the studies of synaptic protein stability indicate a half-life in the order of about one day or less, at least in neuronal cultures (Daly & Ziff, 1997). Thus, the proteins must have increased stability during transport, or else some may reach their destinations already unfolded (this is important especially for neuromuscular junctions, NMJs, which may be meters away from their respective cell bodies).

The transport of soluble proteins along with chaperones (step 7) may help in this case. The chaperones can be buffered by the surface of the SV precursor, and may encounter unfolded proteins, which they bind strongly. The repair reaction would then take place. However, this issue is not well understood. Are there any specific “silent” states for the transport period, so that the proteins do not get modified and damaged? Is SV protein degradation strictly dependent on damage incurred during SV recycling? What could such damage consist in? An interesting alternative answer would be that many of the SV and other proteins do indeed reach the synapse damaged – perhaps resulting in a large fraction of silent, non-recycling vesicles.

### 11 Activity during transport

Too little known to provide a reaction table.

Vesicle precursors cannot “know” they are being transported towards the synapses – and therefore they do not “know” they are supposed to patiently wait for delivery. Do they fuse with each other during transport?

Fusion in the secretory pathway depends on the assembly of four SNARE domains stemming from the two different organelles that are about to fuse. The SNAREs form a 4-helix bundle that draws the two membranes together and forces their intermixing and collapse (Fasshauer *et al*, 1998) (see for example Gao *et al*, 2012; Stein *et al*, 2009 for details on the SNARE zippering mechanism). After fusion, the SNARE bundle is opened up by NSF, an AAA-ATPase that also requires the cofactor α-SNAP (or β-SNAP (Burgalossi *et al*, 2010)) in order to find the SNAREs (Jahn & Scheller, 2006).

Vesicle precursors are likely to be loaded with a variety of SNARE fusion proteins, including syntaxin 1 (Qa) and SNAP-25 (which contains two SNARE domains, Qb and Qc), since these are ubiquitously expressed in virtually all neuronal secretory membranes (Takamori *et al*, 2006). They may also contain some amounts of the highly abundant synaptobrevin (R), and presumably also syntaxin 4 (Qa) or SNAP-23 (Qb-Qc), all of which are molecules involved in the fusion of cargoes to the plasma membrane (Jahn & Scheller, 2006).

Thus, the precursor vesicles do contain the minimal machinery for fusion. As long as the vesicles are in motion they will be unable to dock to each other and fuse. But we have already alluded (step 6) to the formation of bundles of precursor vesicles along the axon. Would the bundled vesicles fuse to each other? They might, but this would be rather irrelevant – it would not make the precursor vesicles any better or worse, from the point of view of SV recycling.

A more complex problem is fusion to the plasma membrane. This should not happen, as it would have serious consequences: the intermixing of cargo with the membrane of the axon, and substantial energy loss (due to the subsequent retrieval of molecules). SNAREs such as syntaxin 1 and SNAP-25 are present in high numbers along the axon (Punge *et al*, 2008; Ribrault *et al*, 2011), perhaps about as much as at active zones (Holderith *et al*, 2012), so again the minimal conditions for fusion are met. As long as the vesicles are in motion, they will not fuse – but what prevents them from docking to the axon? SNAREs themselves do not participate in docking (Geumann *et al*, 2008), but it is unclear whether this explanation is sufficient. It is possible that the SNAREs along the axon are less fusogenic, held in clustered forms that do not participate in SNARE complexing (Sieber *et al*, 2007; Bethani *et al*, 2009; Lang & Rizzoli, 2010). However, this phenomenon remains somewhat of a puzzle. Note that the fusion along the axon is not a mere hypothesis: it takes place abundantly in immature axons. For example, the removal of the cell adhesion molecule NCAM (a controller of synaptic maturation) results in ample exocytosis and vesicle recycling along the axon (Polo-Parada *et al*, 2001; Ryan, 2001).

### 12 Coming off the tracks (Fig 1I)

**Table:** 

Reaction	Unbinding of precursor vesicle from the microtubule, and recruitment to the synapse.
Partners	Precursor vesicle (mobile), and synaptic structures (less mobile; poorly defined).
The trigger	Unclear.
The control mechanism	Unclear. Competition between binding of precursor vesicles to motors, on one side, and to synaptic elements, on the other side? See Wu *et al*, 2013 for a recent description of several molecules involved in the balance between transport and synaptic delivery.

Once the precursor vesicle reaches the end of the microtubule, it will presumably fall off, disengaging from the motor proteins. Of course, this is only relevant for the delivery of the precursor to a synaptic bouton that contains the ends of the microtubules. This, however, is not always the case: many of the *en passant* boutons in the CNS find themselves along the axons, with microtubule bundles passing through the boutons. This is also the case for many NMJs, where the boutons are organized in series. How does then the cargo “know” where to come off?

One explanation may be the transport of precursor vesicles via interactions with Rab3 (see step 6 (Niwa *et al*, 2008)). The sequence of binding events that favors association to the motor favors active, GTP-associated Rab3, over the inactive, GDP-associated Rab3. Perhaps Rab3 exchanges GTP for GDP at synapses, and thus the whole cargo comes off the motor protein (Niwa *et al*, 2008). It is unclear whether this explanation is sufficient. Is Rab3 found predominantly in a GDP-associated form in synapses? Rab3 in synapses is strongly bound to SV membranes in synapses – and thus probably GTP-associated (Fischer von Mollard *et al*, 1990, 1991), since GTP-, but not GDP-associated Rab proteins are thought to bind membranes tightly (Mizuno-Yamasaki *et al*, 2012). Thus, while the GTP/GDP exchange mechanism is a potential trigger for the loosening of the motor/cargo association in the case of Rab3, it is unclear whether this exchange is actually promoted in the synapse. At any rate, this mechanism cannot answer for all SV proteins, since they use different cargo vesicles, as already discussed above (steps 4 and 5). A beautiful recent study of vesicle delivery into synapses of *C. elegans* has revealed the involvement of several other molecular mechanisms, involving the JNK kinase pathway and the G protein ARL-8 (Wu *et al*, 2013).

A simpler hypothesis is that the precursor vesicles do indeed fall off preferentially at microtubule ends. Such ends would then have to be present then in every synapse – which is quite possible even for synapses found along axons. Alternatively, the material may be preferentially unloaded at the end of the microtubule bundle, and may later be shared among the synapses through either diffusion in the cytosol, diffusion in the plane of the membrane or active retrograde transport along the same microtubules (Darcy *et al*, 2006; Fernandez-Alfonso & Ryan, 2008; Westphal *et al*, 2008; Opazo *et al*, 2010; Staras *et al*, 2010). In favor of this hypothesis, distal boutons, found at the end of the elongated *Drosophila* larval neuromuscular junctions, are more active than other boutons found along the axon (Peled & Isacoff, 2011). This could be interpreted as an indication for preferential delivery of some elements, such as SVs or active zone packets, to the end of the axon. Finally, a somewhat similar hypothesis has been made for the transport of neuropeptide-loaded dense-core vesicles from *Drosophila* neurons (Wong *et al*, 2012): these vesicles appear to unload largely within the distal bouton, and to be then retrogradely trafficked towards the cell body, followed by renewed anterograde trafficking. Sporadic capture events place the vesicles in different boutons along the way, both during retrograde and anterograde traffic (Wong *et al*, 2012).

Competing buffering interactions may provide an explanation for this type of sporadic capture (as depicted in Fig 1I). The SV proteins interact with the motor proteins, but also have a strong tendency to interact with proteins of the active zone (synaptic release site). As long as the precursor vesicles are in the axon, the latter tendency is irrelevant. But when passing through a synapse, the binding to active zone components competes with the binding for motor proteins, and may deliver the precursor vesicle to the synapse. The active zone proteins involved in this process may include large proteins known to interact with many substrates, such as bassoon or piccolo (Garner *et al*, 2000), but which do not participate in exocytosis (Mukherjee *et al*, 2010). Alternative interactions may be with the SV clusters, for example, through synapsin, a protein associating with vesicles and with the actin cytoskeleton (Cesca *et al*, 2010).

## In the synapse: forming the first synaptic vesicle

### 13 Putative fusion of the precursor vesicle to the plasma membrane

Reaction table similar to that for step 2.

The precursor vesicle has thus just been delivered to the synapse. It now needs to place the proteins in the right location for forming SVs. Since the precursor is no longer in directed motion along the microtubule, the synapse may be the first optimal place to dock to a membrane and fuse. But where will the precursor vesicle dock and fuse, and to what?

As indicated above (step 11), there are probably sufficient SNAREs in the precursor organelles. They would be fairly fusogenic – indeed, it is unclear why they should not fuse with the axonal membrane. Will these organelles tend to fuse with synaptic vesicles, with synaptic endosomes or with the plasma membrane? SNAREs do not encode for the specificity of fusion (step 11) – it is docking and tethering complexes that do (see, for example, Mills *et al*, 1999) for a review of endosome or carrier vesicle fusion, or Jahn *et al*, 2003 for general membrane fusion). It is still unclear which docking and tethering complexes will work on these organelles, although these mechanisms must be wide-ranging, as the precursor organelles vary substantially in composition (Okada *et al*, 1995). There is much to choose from, since synapses contain a variety of SNAREs and membrane-organizing Rab proteins (Rizzoli *et al*, 2006; Takamori *et al*, 2006). It is possible that the precursor vesicles cannot fuse to SVs as long as these are covered in Rab3 and synapsin molecules (see step 12 for Rab3, step 27 for synapsin). Their potential fusion to endosomes is unclear, especially since the synaptic endosome is one of the least understood cellular organelles.

As for the fusion of the precursor to the plasma membrane, it may happen in constitutive fashion, unrelated to normal SV exocytosis. According to one possibility discussed in step 12, the active zone tethering machinery may even be involved in keeping the precursor vesicle in the synapse, and may therefore also position it in the vicinity of the membrane, favoring the subsequent fusion event between the two organelles. SNAREs such as syntaxin 4 and SNAP-23, together with the highly abundant synaptobrevin, may be the fusion effectors (as in other cell types (Ishiki & Klip, 2005)). The areas of the plasma membrane in which fusion occurs could be different from the active zones used in exocytosis, since syntaxin 4 and the exocytotic syntaxin 1 appear to prefer different plasma membrane areas (Sieber *et al*, 2006).

### 14 Diffusion in the plasma membrane

Reaction table similar to that for step 1.

After fusion to the plasma membrane, the components of the newly fused organelle will segregate in this environment – except for those that remain bound to a common set of partners, and therefore remain in the same membrane domain. Typical plasma membrane components such as syntaxin 4 and SNAP-23 may segregate from the SV components and get stabilized in plasma membrane domains poor in SV components. The SNARE-separating activity of NSF and α- or β-SNAP would be required, especially as it has already been demonstrated that sorting is stopped if SNAREs are not separated (Barysch *et al*, 2009).

It is likely that the lipids of the precursor vesicle could diffuse rapidly away (Zenisek *et al*, 2002) (see Wenk & De Camilli, 2004 for an overview of the lipids involved). However, much of the proteolipid organization may persist as a scaffold for further interactions (see also steps 1, 3).

### 15 Sorting of SV components in the plasma membrane

Reaction table similar to that for step 1.

For the first time since they were generated, the SV proteins find themselves in a membrane that contains a plethora of other SV proteins. A significant fraction of the SV proteins are on the plasma membrane at all times, ranging between 2% and 20% of the total amounts present in synapses (see, for example, synaptobrevin (Sankaranarayanan & Ryan, 2000); synaptotagmin (Opazo *et al*, 2010; Wienisch & Klingauf, 2006); synaptophysin (Granseth *et al*, 2006); endosomal SNAREs (Hoopmann *et al*, 2010); VGLUT1 (Balaji & Ryan, 2007)).

This enables a better organization of the newly fused SV precursor patch. This patch provides an energetically favorable environment for SV proteins, since it already contains high amounts of SV proteins and is probably rich in cholesterol and synaptophysin (see steps 1–3 and Takamori *et al*, 2006). The diffusion of additional SV proteins into this patch is very likely. For example, assuming that the SV precursor patch lacks synaptotagmin: such molecules would immediately enrich within the patch, coming from the neighboring plasma membrane and becoming stabilized there by interactions with cholesterol or with SV proteins such as synaptophysin and synaptobrevin (Bennett *et al*, 1992).

The special environment of the SV patch may indeed depend on cholesterol and on synaptophysin. Since synaptophysin interacts with cholesterol (Thiele *et al*, 2000) and with a variety of SV proteins (Bonanomi *et al*, 2006), it may stabilize the patches of SV material in the plasma membrane. It may also induce (or favor) membrane curvature (as suggested by Thiele *et al*, 2000), adding an additional element to make the fused patch of SV material unique. Although we often use the concept of “vesicle collapse” into the plasma membrane, it is not certain that it has indeed been demonstrated that under normal circumstances the vesicle actually completely flattens onto this membrane. The classic experiments of Heuser and collaborators (Heuser *et al*, 1979; Heuser & Reese, 1981; Miller & Heuser, 1984) show fused vesicles persisting as membrane indentations (at least to some extent) between fusion and endocytosis: these indentations are at first fairly deep, then rather shallow, and eventually deep again, as the clathrin coat forms (see step 17). These indentations, especially if they contain a highly specialized synaptophysin/cholesterol mixture, may favor the permanence of SV proteins within the vesicle patch on the membrane. Arguments against this hypothesis are provided by the fact that synaptophysin-lacking mice are viable and form synaptic vesicles (Eshkind & Leube, 1995; McMahon *et al*, 1996), and that *C. elegans* lacking all synaptophysin-like (tetraspan) proteins are also normal (Abraham *et al*, 2006). It is still possible that the tetraspan proteins have a different influence on cellular processes in *C. elegans*, since these organisms do not contain high levels of cholesterol (Kurzchalia & Ward, 2003), one of the potential interaction partners of tetraspan proteins.

### 16 Recruiting cofactors

Reaction table similar to that for step 4.

The local accumulation of proteins that recognize and bind to SV components will necessarily follow. The vesicle patch will provide a starting structure that allows for the recruitment of proteins that bind its components. For example, synaptobrevin will be recognized by endocytosis adaptors AP180 and clathrin-assembly-lymphoid-myeloid-leukemia (CALM) (Koo *et al*, 2011). Synaptotagmin will be targeted by endocytosis adaptors such as AP2μ and stonin 2 (Diril *et al*, 2006). Some of the neurotransmitter transporters may also be targeted by AP2, via signals discussed above (step 4 (Jung & Haucke, 2007)).

But why are SV proteins recognized with higher affinity in the plasma membrane than in their vesicular form? One possibility is that SV cargo recognition by AP2 is regulated by the presence of the plasma membrane lipid phosphatidylinositol-(4,5)-bisphosphate (PIP_2_) (Cremona & De Camilli, 2001; Höning *et al*, 2005), which increases the affinity of the interaction of AP2 with the cargo. Note, however, that perturbations of individual AP2 components or Stonin do not completely prevent SV formation and recycling (Gu *et al*, 2008, 2013; Kim and Ryan, 2009a,2009b), although they may impair the fidelity of the process (Willox & Royle, 2012; Kononenko *et al*, 2013).

The presence of the endocytotic cofactor proteins in the synapse is probably due to their being buffered by proteins in the SVs (this process is explained in detail under step 42).

### 17 Forming a clathrin-coated vesicle

Reaction table similar to that for step 4.

The initial accumulation of endocytosis adaptors triggers further cascades that will eventually lead to the formation of a clathrin-coated vesicle. I only provide a very brief overview of this process, as many excellent reviews have already covered it in detail (for example Haucke *et al*, 2011; McMahon & Boucrot, 2011).

Adaptor proteins accumulate as in step 16, by binding to SV proteins, which may trigger the further sorting of SV components into the SV patch from the neighboring plasma membrane (step 15). This is followed by the bending of the plasma membrane locally by, for example, cofactor proteins that insert amphiphatic helices into the intracellular face of the membrane (including proteins such as amphiphysin and endophilin; see also McMahon & Gallop, 2005). The nascent vesicle is then covered by a clathrin coat composed of clathrin assemblies containing three light and three heavy chains of the clathrin molecule, termed “triskelia”.

Although this scheme has often been described and is entirely convincing, it is rather difficult to understand how the recruitment of all of the components can be effected with the efficiency that this process implies. The different cofactors are recruited in a clear temporal sequence (Taylor *et al*, 2011; Cocucci *et al*, 2012) – but how do they “know” where to get recruited? AP2 may be more inclined to bind to synaptotagmin when the latter is in the PIP_2_-containing plasma membrane, rather than in vesicles (step 16), but how is it that other proteins that interact with AP2 will only now be recruited to AP2 itself? Also, endophilin and amphiphysin may well be recruited to a “bump” of vesicle material on the plasma membrane, given their natural tendency to bind to such areas, but why do these proteins not assemble elsewhere as well? Endophilin binds to the curved membranes of SVs (Bai *et al*, 2010) – why does it leave them to actually enrich on the endocytotic vesicle; do changes in the phospholipid concentrations determine such recruitment events? (Posor *et al*, 2013).

One possible answer is suggested by the fact that endocytosis is a localized event, where multiple cofactors are recruited through a buffer effect (according to the framework proposed above). The sequence of events may be the following one: synaptophysin and cholesterol form a stable basis for the SV. This is probably also complemented by other proteins such as the glycosylated synaptotagmin and SV2, whose intravesicular glycan chains may bind to each other to form the fairly compact intravesicular structure observed in frozen, freeze-substituted NMJs (Heuser & Reese, 1981; Harlow *et al*, 2013). Upon fusion, the interactions of synaptophysin, cholesterol and possibly glycans allow the vesicle to remain complete, by and large, possibly in the form of a dimple on the membrane (see step 15). This structure is bound by BAR-domain proteins (Galic *et al*, 2012) and afterwards by other adaptor molecules (step 16). When such adaptor molecules are recruited elsewhere, such as to SV proteins in the synapse, their recruitment only achieves low-affinity interactions, resulting in the buffering of individual adaptor molecules but not in their stabilization in the form of a coat. The stronger (higher-affinity) interaction provided by the fused vesicle recruits many more adaptors simultaneously, permitting the eventual accumulation of clathrin molecules. Finally, clathrin accumulation results in a stable and rigid structure that allows the completion of the coating reaction.

### 18 Triggering endocytosis (Fig 2A)

**Table:** 

Reaction	Binding of budding cofactors onto the SV protein/lipid patch on the plasma membrane.
Partners	Soluble budding cofactors (including coat proteins and their adaptors), and SV proteins and/or lipids arranged in a patch (domain) on the plasma membrane.
The trigger	At the moment unclear. Possibly the entry of calcium into the synapse after action potential activity (Yao *et al*, 2009). Or exocytosis, by unknown mechanisms. Calcium may be the primary trigger, since high calcium entry results in a strong increase of endocytosis, raising it above the amount of exocytosis (endocytosis overshoot (Xue *et al*, 2012)). The effect of calcium on actually slowing endocytosis, observed in hippocampal cultures, does complicate this interpretation (Leitz & Kavalali, 2011).
The control mechanism	The budding cofactors are kept within the synapse by interactions with the vesicle cluster, which acts as a buffer for these proteins (Denker *et al*, 2011). The size of the vesicle cluster (vesicle pool) thereby controls the amounts of budding molecules, and therefore controls this reaction. Calcium may trigger the unbinding of the proteins from the vesicle cluster (Denker *et al*, 2011), or may interact with calcium sensors such as calmodulin (Wu *et al*, 2009).

An important question in this process is what triggers the endocytosis event. An assumption has been that it is triggered by the insertion of the newly released vesicular membrane into the plasma membrane (Ceccarelli & Hurlbut, 1980). This idea is in line with most of the classical electron microscopy observations of membrane recycling – indeed, no endocytosis events could be observed without strong stimulation and the ensuing exocytosis (Heuser & Reese, 1981). However, a number of more recent observations have challenged this assumption. For example, monitoring exo- and endocytosis with single-vesicle sensitivity also revealed that synapses occasionally responded to stimulation by selectively endocytosing SVs, rather than by releasing them (Gandhi & Stevens, 2003).

Much has been discussed in terms of stimulation directly triggering endocytosis (Cousin & Robinson, 1999, 2001; Clayton *et al*, 2007). Extracellular calcium appears to be essential (Henkel & Betz, 1995; Zefirov A *et al*, 2006), and specialized endocytosis-coupled calcium channels may even be involved (Kuromi *et al*, 2004; Yao *et al*, 2009). The calcium influx may activate the Ca^2+^-dependent phosphatase calcineurin, resulting in the dephosphorylation of several endocytosis cofactors, including dynamin, amphiphysin or AP180 (Clayton *et al*, 2007). These proteins are later phosphorylated by kinases such as CDK5, and both events may be important for endocytosis (Evans & Cousin, 2007). This was confirmed also by the fact that calcineurin and CDK5 have opposing effects on the amount of vesicles that recycle in cultured synapses: calcineurin knock-down reduces the proportion of recycling vesicles, while CDK5 increases it (Kim & Ryan, 2010). Various other proteins may also be involved, such as calcium sensors (for example, synaptotagmin, calmodulin (Igarashi & Watanabe, 2007)).

Overall, endocytosis does appear to require extracellular calcium (and stimulation to bring it into the synapse). Some evidence points to a specific endocytosis sensor that is activated upon stimulation. A different explanation for the need for calcium is offered by the hypothesis that many of the soluble cofactors needed for endocytosis are found at rest in relatively immobile states, bound to synaptic vesicles or to some other synaptic structure (Shupliakov, 2009; Denker *et al*, 2011, 2011) (see step 42). This suggests that at rest only a handful of endocytotic cofactors are available, and therefore fused SV patches cannot be retrieved. They may engage some of the cofactors, but cannot proceed further, due to a lack of soluble cofactors. The entry of calcium changes the interaction of the soluble proteins with the vesicles, perhaps in the manner of a simple, electrostatic interaction (Zilly *et al*, 2011), and liberates them (Denker *et al*, 2011). The newly freed cofactors can now diffuse to the SV patches and complete the endocytosis process. This is a much simpler and perhaps more effective regulation of endocytosis by calcium – although it is highly speculative at this point.

### 19 Severing the vesicle from the plasma membrane

Reaction table similar to that for step 4.

The GTP-ase dynamin is recruited to the site of endocytosis, through a variety of interactions with cofactors that have already been brought there (steps 16–17), including amphiphysin, endophilin and intersectin (Haucke *et al*, 2011). Intersectin is an important scaffolding molecule in endocytosis (Pechstein *et al*, 2010), and may be involved in organizing the site at which the vesicle will be pinched off the plasma membrane. A ring-like dynamin assembly forms around the “neck” of the endocytosing vesicle (for example, Takei *et al*, 1996), and is clearly involved in pinching the vesicle off the membrane, although the precise mechanisms are still debated (Faelber *et al*, 2012). As for other endocytosis cofactors (steps 16–18), it is likely that dynamin is maintained in the synapse by buffering interactions with some synaptic elements, such as the synaptic vesicles.

## In the synapse: fine-tuning to obtain a perfect synaptic vesicle

### 20 Involvement of actin in endocytosis

Poorly known reaction, despite extensive research on synaptic actin.

The coated vesicle just liberated from the plasma membrane is now able to diffuse away. However, much evidence suggests that it does not do so: as observed in other types of cells as well, the newly endocytosed vesicles appear to be propelled within the cells by actin polymerization (Merrifield *et al*, 1999). The actin growth that accompanies endocytosis appears to propulse the vesicles back onto the vesicles clusters (Shupliakov *et al*, 2002; Bloom *et al*, 2003). As suggested in recent reviews (Haucke *et al*, 2011), the cause of the activity of actin is unclear. There is no obvious reason why the newly endocytosed vesicles should be actively pushed into the synapse, since these vesicles are mobile and can simply diffuse within the synapse (Gaffield *et al*, 2006; Westphal *et al*, 2008; Kamin *et al*, 2010).

An alternative explanation is that actin is recruited to assist with membrane tubulation (invagination) during endocytosis, just before the dynamin-mediated scission (Ferguson *et al*, 2009). In this case the actin push may liberate the endocytotic sites for the formation of new vesicles. The liberation of the active zone, to allow for the fusion of new vesicles, is indeed a convincing bottleneck in synaptic recycling (Kawasaki *et al*, 2000; Hosoi *et al*, 2009; Neher, 2010). But is actin really necessary to move the fused SV patch from the active zone, or is actin only involved during the process of pinching off the vesicle?

Answers to this question are still vague. At any rate, the perturbation of endocytosis is the most consistent observation made upon the addition of actin depolimerizing drugs (Rizzoli & Betz, 2005), despite the lack of sufficient explanations for its action.

### 21 Letting go of actin and uncoating (Fig 2B)

**Table:** 

Reaction	Uncoating of clathrin-coated vesicle.
Partners	Clathrin-coated vesicle (with relatively low mobility), and soluble uncoating factors (auxilin, Hsc70).
The trigger	Separation of coated vesicle from plasma membrane, exposing the “hole” in the coat.
The control mechanism	Not fully clear. How are sufficient amounts of auxilin and Hsc70 maintained in the synapse? Do they spontaneously reach the coated vesicle? Does the coated vesicle act as a buffer for some auxilin and Hsc70 molecules, which constantly probe its surface, and thus are maintained in its vicinity?

After penetrating into the synapse for a limited length, the actin filaments presumably stop and depolimerize. It is unclear how this happens: when does actin polimerization stop, and why?

Soon after the scission event (step 19) the vesicle starts uncoating. The area of the coated vesicle where the “neck” severed by dynamin was located is, after scission, freely accessible, and also free of clathrin. This area is likely to be recognized with high affinity by two molecules involved in disassembling the coat, Hsc70 (also discussed under step 7 above) and its cofactor auxilin, since the imperfect coverage of clathrin seems to favor the binding of these molecules (McMahon & Boucrot, 2011). Additionally, interactions between dynamin and auxilin may recruit the latter to the coated vesicle (Newmyer *et al*, 2003; Sever *et al*, 2006). The mechanisms of uncoating rely on the ability of Hsc70 to bind to and destabilize numerous clathrin heavy chains simultaneously, which results in the loss of the coat structure (Xing *et al*, 2010; Böcking *et al*, 2011). An interesting twist to this story is that endophilin (discussed above as a membrane-bending protein) may recruit during endocytosis the enzyme synaptojanin, which dephosphorylates PIP_2_ (Cremona *et al*, 1999; Milosevic *et al*, 2011) and may thus promote uncoating.

### 22 The new vesicle does not fuse to SVs

Reaction scheme similar to that of step 4.

The newly formed SV would now, in principle, be able to fuse with other organelles, including other SVs. The SVs contain all the SNARE molecules necessary for fusion events (Takamori *et al*, 2006); and the newly endocytosed vesicles even appear to have increased amounts of the plasma membrane SNAREs syntaxin 1 and SNAP-25 (Hoopmann *et al*, 2010). However, they do not appear to ever fuse homotypically to any great extent (see, for example, Murthy & Stevens, 1998). Why?

This could be due to a lack of SV-to-SV docking factors. Endosomes and carrier vesicles, for instance, need to contain the appropriate membrane-organizing Rab molecules to generate domains able to tether to each other in order to promote fusion (for example, Zerial & McBride, 2001). But would this be a problem in the synapse? The concentration of vesicles is extremely high, and it would not be difficult for the SNAREs to interact with each other.

Alternatively, perhaps the vesicles are kept in a non-fusogenic state by being caged by synapsin or other molecules that cross-link vesicles and link the vesicles to the cytoskeleton (Hirokawa *et al*, 1989; Siksou *et al*, 2007; Fornasiero *et al*, 2012). Indeed, the average SV is extremely limited in its movement (Jordan *et al*, 2005; Lemke & Klingauf, 2005; Shtrahman *et al*, 2005; Yeung *et al*, 2007) and it is possible that it is covered in synapsin molecules that it cannot really fuse to any other membrane. Another molecule that may coat vesicles and remove their possible homotypic fusion is their main Rab molecule (Rab3). However, no defects pointing to aberrant homotypic fusion of SVs to other SVs were detected in Rab3 knock-outs – this molecule is more likely to be involved in priming the vesicles for exocytosis (Schlüter *et al*, 2004, 2006).

### 23 Fusion to synaptic endosomes

Poorly known. Presumably similar to the fusion of carrier vesicles to Golgi (see step 2).

Although the influence of endosomes in the synapse has been strongly debated, several lines of evidence indicate that newly endocytosed vesicles fuse to early or sorting endosomes. The membrane organizer of early endosomes, Rab5, is present on synaptic vesicles (Fischer von Mollard *et al*, 1994), and its perturbation causes abnormal organelles to be present in axons (De Hoop *et al*, 1994). Since Rab5 is also implicated in endocytosis, however, these abnormalities cannot be taken as proof of the involvement of endosomes in the vesicle cycle. However, recent work has provided substantial evidence in favor of the endosomal sorting of SV components: synaptic endosomes change in morphology upon synapse stimulation in a fashion suggesting SV recycling (Wucherpfennig *et al*, 2003). Abnormal endosome-like intermediates appeared upon perturbing the dynamics of PI(3)P, the phosphoinositide class most often connected to endosomes (Rizzoli & Betz, 2002). Finally, synaptic vesicle recycling was affected when molecules involved in endosomal fusion (Hoopmann *et al*, 2010) or sorting (Uytterhoeven *et al*, 2011) were perturbed.

Therefore, it is currently thought that the newly endocytosed vesicle represents a target for endosomal fusion. This may take place through the generation of PI(3)P on the surface of the newly uncoated vesicle, which recruits PI(3)P-binding proteins, and eventually the membrane organizer Rab5. As PI(3) kinases count among the effectors of Rab5 (Di Paolo & De Camilli, 2006), the reaction snowballs and may recruit enough endosomal cofactors to transform the newly endocytosed vesicles into an endosome-like organelle able to undergo homotypic fusion with *bona fide* endosomes (Hoopmann *et al*, 2010).

### 24 Endosomal sorting

Poorly known. Presumably similar to sorting in the Golgi (see step 3).

Although it has been suggested (and even shown to some extent (Hoopmann *et al*, 2010)) that the endosome does contribute to the sorting of contaminant molecules from the vesicles, the nature of this process is still difficult to understand. It is tempting to assume that the molecules that organize the structure of the vesicle, including synaptophysin and cholesterol (as already noted under steps 1–5), continue to do so in the endosome.

Why would the mechanisms for vesicle formation need to be refined in the endosome? Perhaps the endosome contains a variety of binding opportunities and lipid environments that offer new sorting opportunities, different from those of the plasma membrane. The two membranes (endosome, plasma membrane) are quite different, in terms of both composition (they contain, for example, different levels of phosphoinositides) and physical bilayer characteristics (Sharpe *et al*, 2010).

### 25 Endosomal budding

Poorly known. Presumably similar to other budding steps (for example, step 4).

The lack of information on how endosomal sorting takes place in synapses also affects our understanding of the budding of SVs from the endosome. Despite early suggestions that clathrin may be involved (Heuser & Reese, 1973), this is by no means clear. Clathrin may reform SVs from plasma membrane infoldings, or from plasma membrane-derived vacuoles (Takei *et al*, 1996; Kasprowicz *et al*, 2008), but these organelles are by no means equivalent to *bona fide* endosomes. One possibility has been suggested by investigations of small-vesicle formation in the neuroendocrine PC12 cell line. Here the adaptor complex AP3 may act as a coat to form such vesicles specifically from endosomes, probably in interplay with the ADP ribosylation factor 1 (ARF1), a protein likely involved in recruiting AP3 (Faúndez *et al*, 1997, 1998). One key SV molecule recognized by these adaptors may be synaptobrevin (Salem *et al*, 1998).

Although PC12 vesicles are not identical to synaptic vesicles, the endosomes forming them can fuse to synaptic organelles (Rizzoli *et al*, 2006), and do contain most of the SV molecules. In addition, AP3 has been implicated in synaptic vesicle recycling in neurons (Voglmaier *et al*, 2006), which suggests that this is indeed a potential pathway for vesicle formation from endosomes.

### 26 Filling with neurotransmitter (Fig 2C)

Two reactions: acidification and neurotransmitter uptake.

**Table:** 

Reaction	Acidification of the vesicle.
Partners	vATPase on the vesicle membrane (low mobility), and protons in the synapse (high mobility).
The trigger	Spontaneous interaction of ATP and proton with the vATPase?
The control mechanism	Unlike many other mechanisms indicated above, this reaction may not require any buffering. The ATP and proton concentrations are presumably high enough to ensure that the vATPase meets them at any time, without the need for buffering. Also, their small size and high mobility may make their retention in the vicinity of the vATPase (buffering) inefficient.

**Table:** 

Reaction	Neurotransmitter entry into the vesicle.
Partners	Neurotransmitter transporters on the surface of the vesicle (low mobility), and protons within the vesicle, neurotransmitter molecules in the cytosol (high mobility).
The trigger	Spontaneous interaction of protons and neurotransmitter molecules with the neurotransmitter transporters?
The control mechanism	Similar to Reaction 1 above. May not require any buffering phenomenon.

The processes discussed so far have finally resulted in the formation of a well-sorted, fusion-competent SV. It is likely that it is already filled with neurotransmitter, relying on the exchange of protons from the vesicle lumen for neurotransmitter molecules from the cytosol. This has been demonstrated for monoamine or acetylcholine transport, which involve the exchange of one transmitter molecule for two vesicular protons (antiport) (Edwards, 2007). Glutamate transporters (VGLUT) may not rely on this relatively simple antiport, but are nevertheless dependent on the SV acidification (Ahnert-Hilger *et al*, 2003; Edwards, 2007; Omote *et al*, 2011). In addition, their regulation by chloride ions is still under intense investigation (Juge *et al*, 2010) (see also Goh *et al*, 2011 for further details on the regulation of glutamate filling into synaptic vesicles by K^+^/H^+^ exchange). The vesicular proton concentration is maintained by a proton pump (vATPase; Saroussi & Nelson, 2009), which is probably recruited to vesicles by interactions with synaptophysin (Galli *et al*, 1996) or synaptobrevin (Di Giovanni *et al*, 2010). The enzymatic activity of the vATPase may be unrelated to the molecule's presence within vesicles, since it continues in the plasma membrane of the nerve terminal, after exocytosis (Zhang *et al*, 2010).

SV acidification, measured by imaging the quenching of pH-sensitive GFP molecules coupled to the luminal domain of synaptic proteins (pHluorins; Miesenböck *et al*, 1998) may be as fast as a few hundreds of milliseconds after the vesicle is disengaged from the plasma membrane (Gandhi & Stevens, 2003). The speed of this reaction, however, may have been overestimated (Granseth *et al*, 2006). The refilling with neurotransmitter molecules, recently investigated by electrophysiological experiments combined with photolysis of caged glutamate, seems substantially longer, with a time constant of approximately 15 s (Hori & Takahashi, 2012).

After the vesicles are refilled, they seem to remain relatively inert in terms of neurotransmitter exchange. This has mostly been discussed in the context of replacing native neurotransmitters with modified ones, and observing the incorporation and/or release of the latter. Despite several controversies, the overall conclusion is that once they are filled, the vesicles do not receive any more neurotransmitter molecules, unless they fuse, release their contents, and thus require refilling (Van der Kloot, 2003).

## Neurotransmitter release

### 27 Vesicle mobility (Fig 2D)

**Table:** 

Reaction	Interaction of the newly uncoated vesicle with synapsin molecules.
Partners	SV (mobile), synapsin molecules in the vesicle cluster (far less mobile).
The trigger	Spontaneous interaction of the vesicles with synapsin molecules?
The control mechanism	The synapsin molecules are maintained in the synapse by interactions with the SV cluster, in the same fashion in which the abundance of buffering cofactors is controlled (step 18). The control over which SV interacts with synapsin molecules (and is captured into the SV cluster) is unclear: differences in SV composition? Random interactions with synapsin molecules?

In contrast to most other vesicles, the newly formed SVs are remarkably mobile (Gaffield *et al*, 2006; Kamin *et al*, 2010). One possible explanation for this is that newly formed SVs lack synapsin. Synapsin seems unable to bind to coated vesicles and is thus shed during recycling (Bloom *et al*, 2003). It may also be not be able to bind SV molecule patches on the plasma membrane as it prefers binding to the curved surfaces of the SVs (Krabben *et al*, 2011). At rest, the newly recycled vesicles are able to move, and their mobility remains unchanged by physiological stimuli (Gaffield *et al*, 2006; Kamin *et al*, 2010). When the stimulus surpasses physiological levels, the movement of other, normally immobile SVs (Gaffield *et al*, 2006) is triggered, by calcium-triggered phosphorylation of synapsin, which causes the unbinding of the molecule from vesicles, and their subsequent liberation from vesicle clusters (see reviews Cesca *et al*, 2010; Hilfiker *et al*, 1999). This view has been challenged by conflicting observations on synapsin-null mutants. For example, synapses from mice lacking synapsin still contained filament-like connections between the synaptic vesicles (Siksou *et al*, 2007), and their vesicles appeared as mobile as the wild-type vesicles (Gaffield & Betz, 2007). However, the phenotype of mice lacking synapsin is difficult to interpret, as, for example, they contain far fewer vesicles, which complicates any measurements (Fornasiero *et al*, 2012). In *Drosophila* the interpretation is much easier: the boutons lacking synapsin contain ample amounts of SVs, which are significantly more mobile than their wild-type counterparts (Denker *et al*, 2011).

Experiments in which single vesicles have been monitored, in the presence or absence of stimulation, are dominated by random movements (Westphal *et al*, 2008; Kamin *et al*, 2010; Lauterbach *et al*, 2010; Park *et al*, 2012). Directed, motor-driven movement of SVs within the synapse is probably limited. SVs are exchanged between synapses along the axon in an active fashion, along microtubule and/or actin strands, as mentioned above (step 12; Darcy *et al*, 2006; Westphal *et al*, 2008). But within most synapses the microtubules are simply not in the right place to move the vesicles down to the active zone, and actin also seems not to be required for active SV transport to active zones (Sankaranarayanan *et al*, 2003).

Directed, stimulation-dependent movement of SVs towards the active zone is often described as “making sense”. However, why should this be so? The average active zone is silent most of the time, and when it is active it only releases a few vesicles at one time (Denker *et al*, 2011; Körber *et al*, 2012; Marra *et al*, 2012). It has a large number of vesicles docked (Schikorski & Stevens, 1999, 2001) – note, however, that not all may be ready to fuse (Xu-Friedman *et al*, 2001; Rizzoli & Betz, 2004). Finally, the random motion of the mobile vesicles will always ensure the relatively rapid refilling of the active zone sites that remain empty after vesicle fusion (indeed, clearing these sites of fused vesicles may be a more critical bottleneck in SV recycling than refilling them; Neher, 2010).

### 28 Docking at the active zone (Fig 2E)

**Table:** 

Reaction	Docking of SV.
Partners	SV (mobile), site on the active zone (immobile).
The trigger	Liberation of a docking site on the active zone, by exocytosis of a previously docked vesicle.
The control mechanism	Mobile synaptic vesicles are maintained in the vicinity of the active zone (are buffered in the vicinity of the active zone) by repeated interactions with individual active zone proteins. The liberation of a docking site allows multiple interactions with active zone proteins, which stabilize the vesicle at this site.

One of the mobile vesicles from step 27 may encounter an empty site at the active zone and dock to it. Slotting into the active zone is likely driven by SV binding to Rab3-interacting molecules (RIMs) (Haucke *et al*, 2011) and the RIM-binding proteins (Liu *et al*, 2011). These interactions position the vesicle at the active zone, presumably in the vicinity of the calcium channels. The docked vesicle are thus in an optimal position to sense calcium entry into nerve terminals upon neuronal activity (see step 31). The docking may be initiated by the interaction of the vesicle with other synaptic molecules, such as the scaffolding proteins bassoon (Hallermann *et al*, 2010) and piccolo that may bring the mobile vesicle closer to the RIM molecules (see step 12; see Dani *et al*, 2010 for the positions of the proteins in relation to the active zone).

The docking machinery may contain SNAREs from the SV and from the plasma membrane that bind to each other (synaptobrevin, syntaxin 1 and SNAP-25; Walter *et al*, 2010). Synaptotagmin from the SV likely bind to plasma membrane SNAREs (De Wit *et al*, 2009) or to phosphoinositides in the plasma membrane (see also Stein *et al*, 2007). When observed using electron tomography, the docking machinery appears relatively complex, and may contain several other molecules (Harlow *et al*, 2001; Nagwaney *et al*, 2009; Szule *et al*, 2012). Recent results suggest that the vesicle can only dock in one position at the active zone, with one domain on the vesicle always coming in contact with the active zone machinery (Harlow *et al*, 2013). Unfortunately, the nature of the interacting domains and molecules is not yet clear, although immuno-electron microscopy studies may soon provide some answers (Limbach *et al*, 2011).

The replenishment of the active zone with vesicles, after liberation of active zones, is not well understood. Numerous proteins have been involved in this process, including, for example, the priming protein Munc13 and the calcium-interacting protein calmodulin (Lipstein *et al*, 2013). In addition, the organization of the active zone is critical for the docking and fusion process. Changes in the active zone structure are reflected by changes in SV release (Matz *et al*, 2010). The influence of the active zone on coordinating both exo- and endocytosis has been heavily scrutinized in the last few years. While the precise mechanisms are still unclear, several key proteins have been identified. For example, Rab3, besides its influence on SV dynamics, is also involved in the composition of the active zone (Graf *et al*, 2009), possibly by interactions with active zone proteins such as Bruchpilot (Kittel *et al*, 2006) a homolog of the mammalian ELKS/CAST/ERC. *Bruchpilot* itself may be an important hub of active zone modulation (Miśkiewicz *et al*, 2011). Finally, Rab3-interacting molecules (RIMs) are also vital for the organization of the active zone, by interacting not only with Rab3, but also with calcium channels, which are recruited to active zones by binding to RIMs (Deng *et al*, 2011; Han *et al*, 2011; Kaeser *et al*, 2011, 2012; Liu *et al*, 2011; Graf *et al*, 2012; Müller *et al*, 2012). The mechanisms of RIM interactions with the vesicles may also involve Munc13 (Deng *et al*, 2011), which provides a link between RIM and the mechanisms of SV fusion (see step 30). Finally, endocytosis defects have been observed in cells with perturbed active zones, which suggests that the active zone may affect this process as well (Khimich *et al*, 2005) (see step 33 for further discussion).

### 29 What happens to vesicles that do not dock

Reaction table similar to those for steps 22 and 27.

Mobile vesicles that are not caught by the active zone machinery will remain mobile and may continue to move around the vesicle cluster. Eventually they will bind molecules such as synapsin and lose their mobility (Denker & Rizzoli, 2010; Kamin *et al*, 2010). Synaptic inactivity will increase the proportion of immobile vesicles, since the continual recycling that cleans vesicles of synapsin (step 27) no longer takes place in a synapse that does not recycle vesicles.

### 30 Preparing for fusion (Fig 2F)

**Table:** 

Reaction	Partial complexing of vesicle SNAREs (synaptobrevin) with plasma membrane SNAREs (syntaxin 1, SNAP-25).
Partners	Synaptobrevin molecules on docked vesicle (with relatively low mobility), and syntaxin 1, SNAP-25 molecules on the plasma membrane (far more mobile).
The trigger	Docking.
The control mechanism	The large number of synaptobrevin molecules on the membrane of the vesicle (˜70; Takamori *et al*, 2006) acts as a buffer for syntaxin 1 and SNAP-25 molecules. These interact with the synaptobrevin molecules, and become locally enriched (Barg *et al*, 2010). Triple complexes, containing all three SNAREs, may then form spontaneously.

As suggested at step 28, the vesicle SNARE (synaptobrevin) interacts with, and engages, plasma membrane SNAREs to form partially coiled SNARE complexes (see step 11). This does not imply that the latter need to be enriched at the active zone (see for example, Punge *et al*, 2008; Ribrault *et al*, 2011), as has been suggested in the past. The vesicle's presence at the active zone will be sufficient to capture several plasma membrane SNAREs, according to the framework discussed above. This generates stable, partially coiled SNARE complexes. Apparently only a few such complexes form per vesicle (Mohrmann *et al*, 2010; Van den Bogaart *et al*, 2010; Sinha *et al*, 2011; Shi *et al*, 2012). The partially coiled SNAREs appears to be stabilized by complexin (Jahn & Fasshauer, 2012) that may be buffered by SVs in the synapse, and delivered by the docked SVs at the active zone (Wragg *et al*, 2013) (see also Xue *et al*, 2009; Giraudo *et al*, 2009 for further details on the influence of complexin on synaptic release). Two other important proteins are involved in the SNARE interactions, Munc18 and Munc13, which have been often implicated in vesicle priming and fusion. A beautiful recent *in vitro* study, investigated the interactions between the three SNAREs, the two Munc molecules, synaptotagmin and NSF/α-SNAP, and proposed that Munc18-1 and Munc13 influence fusion mainly by binding to syntaxin 1 to keep it in a conformation that is favorable for SNARE complex formation (Ma *et al*, 2013). In addition, recent works suggest that not only the presence, but also the positioning of the *C. elegans* UNC-13 proteins at the active zone is critical for synaptic release (Hu *et al*, 2013; Zhou *et al*, 2013). Finally, other proteins, such as snapin (Pan *et al*, 2009), may be involved as well in SV fusion.

The involvement in the fusion or docking processes of homotypic SNARE clusters, built by syntaxin 1 (Sieber *et al*, 2007), SNAP-25 (Halemani *et al*, 2010) or even synaptobrevin (Bethani *et al*, 2009) is unclear. They may represent clusters in which the reactivity of the different molecules is reduced (Bethani *et al*, 2009), or, in contrast, may be docking sites for the vesicles. Two observations argue in favor of the latter hypothesis, at least for syntaxin 1. First, syntaxin 1 clusters appear to interact with PIP_2_ (Van den Bogaart *et al*, 2011; Honigmann *et al*, 2013), a molecule that also interacts with SV proteins (synaptotagmin), and therefore is optimally positioned to be part of SV docking. Second, syntaxin 1 clusters colocalize with PI(3,4,5)P3 (Khuong *et al*, 2013), whose reduction inhibits both syntaxin 1 clustering and neurotransmitter release – thus indirectly suggesting that the clusters are important for transmitter release. Nevertheless, the docking of dense-core vesicles to SNARE clusters is not particularly evident in PC12 cells (Barg *et al*, 2010). These vesicles may even prefer areas free of SNARE clusters (Yang *et al*, 2012).

It is unclear why homotypic clusters of SNAREs should exist in the first place. The SNAREs are extremely abundant: 70 copies of synaptobrevin per synaptic vesicle (Takamori *et al*, 2006), and large numbers of both SNAP-25 and syntaxin 1 on the plasma membrane (about 4–5% of the entire protein contents of synapses; Walch-Solimena *et al*, 1995). This, at first glance, would seem a drastic waste of resources, since fusion only requires a few SNARE complexes. However, it is possible that the high abundance of SNAREs is important to the cell for reasons that are not clear yet. Possibly not even for synaptic release; for example, strongly reducing SNAP-25 levels by knock-down approaches does not eliminate exocytosis (Sharma *et al*, 2011). The neurodegeneration caused by reducing SNAP-25 numbers, however (Sharma *et al*, 2011), suggests that the abundance of these molecules is important to the organism, in ways that still remain to be fully understood.

### 31 Fusion (Fig 2G)

**Table:** 

Reaction	Synaptotagmin interaction with plasma membrane lipids.
Partners	Plasma membrane lipids, synaptotagmin on synaptic vesicle.
The trigger	Calcium entry.
The control mechanism	Unclear. Does it need any buffering, as long as synaptotagmin molecules are already provided at the right location by being sequestered in the docked vesicle?

Upon neuronal activity, calcium enters through voltage-gated channels at the active zone and stimulates fusion. Synaptotagmin, the best-known calcium sensor, detects the calcium changes and allows fusion to take place. Although the exact mechanisms are still under discussion, calcium-bound synaptotagmin interacts simultaneously with SNAREs and with the plasma membrane, perhaps destabilizing the bilayers and/or allowing SNAREs to coil fully around each other (completing the partial coil from step 30), and to complete the fusion process (see reviews Jahn & Fasshauer, 2012; Chapman, 2008).

SNARE-mediated fusion has already been discussed above (step 11). It results in the “collapse” of the vesicle into the membrane – although it is quite likely that the flattening of the vesicle into the membrane is not complete (step 15). Only a few SNARE complexes are likely to be necessary for the fusion step (Mohrmann *et al*, 2010; Van den Bogaart *et al*, 2010; Sinha *et al*, 2011).

The response of synaptotagmin to calcium is based on its being in a calcium-free state before neuronal activity. At rest, calcium is buffered by calcium-binding proteins or sequestered in other compartments (ER, mitochondria), while synaptotagmin does not have substantial access to calcium. The situation changes when the calcium concentration rapidly increases in its vicinity (at the active zone) upon neuronal activity.

A fascinating issue in exocytosis has developed during the last few years, with numerous observations confirming that an SV-associated chaperone molecule, CSPα (Takamori *et al*, 2006), is required for the stability of SNAP-25 (Sharma *et al*, 2011), and thereby may control exocytosis. The connection of this protein to dynamin, and therefore to endocytosis (Zhang *et al*, 2012) suggests that it may even be a link between exo- and endocytosis.

### 32 First events after exocytosis

Reaction table similar to those for steps 1, 3, 14 and 15.

The vesicle material may diffuse into the plasma membrane, either as single molecules or as a package. Using super-resolution microscopy, it was suggested that molecules remain clustered upon fusion, to be later targeted by the endocytosis machinery as a cluster (Willig *et al*, 2006; Hoopmann *et al*, 2010; Opazo *et al*, 2010). The rapid diffusion of vesicle molecules out of synapses upon strong stimulation (Li & Murthy, 2001; Fernández-Alfonso *et al*, 2006; Wienisch & Klingauf, 2006) has led to the alternative hypothesis that fused SVs disperse into single, rapidly moving molecules (see also Zhu *et al*, 2009).

However, the fact that the membrane contains a reservoir of SV material that does not intermix with newly exocytosed SVs upon mild stimulation (Wienisch & Klingauf, 2006) has strengthened the conclusion that SV material remains in patches, as originally suggested (Willig *et al*, 2006), and as recently demonstrated by other groups (Hua *et al*, 2011a) as well. As indicated above (steps 14–15), this doesn't discount the diffusion of a few SV molecules into or out of the fused vesicles. However, it is unlikely that a wholesale dispersal of SV molecules takes place, to be followed by their re-grouping through the activity of the endocytosis machinery. Also, according to the framework discussed above, the endocytosis machinery cannot be assembled on single SV molecules – the single, dispersed molecules cannot bring sufficient adaptors around to engage endocytosis. Therefore, it seems hardly possible that the newly fused vesicles should fully disperse, the more so since this would contradict our understanding of other sorting steps based on the self-assembly of a vesicle sub-structure (for example, steps 1–5, 14–17).

### 33 Endocytosis

Reaction table similar to those for steps 4, 16 and 17.

An issue that has received significant attention lately is the connection between exo- and endocytosis (see for example Haucke *et al*, 2011). Much of the current understanding of the effects of endocytosis on subsequent exocytosis comes from an experiment performed with *Drosophila* synapses containing a temperature-sensitive variant of the endocytotic protein dynamin (*shibire*) (Kawasaki *et al*, 2000). During a stimulation train, the *shibire* synapses responded normally to the first action potential, but then released significantly fewer vesicles than wild-type synapses. This could not be attributed to a lack of releasable vesicles (which were still plentiful), but rather to a reduction of exocytosis caused by the inhibition of dynamin, suggesting a strong connection between exocytosis and endocytosis. That being said, the reduction induced by dynamin was not particularly potent. The *shibire* animals continue to release vesicles, for long time – in fact, they can release virtually all of their vesicles (Denker *et al*, 2011). In contrast, blocking endocytosis by peptides that inhibit AP2 or dynamin appeared to counteract exocytosis more strongly in the calyx of Held (Hosoi *et al*, 2009), virtually stopping membrane retrieval. The interpretation was that the endocytosis machinery is required for removing the SV material from the active zone, and clearing it for new fusion events (Neher, 2010). This is in agreement with the notion that endocytosis does not take place at the active zone itself, but in its vicinity (see, for example, Miller & Heuser, 1984).

This hypothesis called for the identification of mechanisms that couple exo- to endocytosis, in addition to their known connection through calcium (which acts as a trigger for both of the processes, as discussed above). One possible connection was provided by the exocytotic SNARE molecules. Enzymatic cleavage of synaptobrevin (by tetanus toxin) blocked (Hosoi *et al*, 2009) or at least slowed (Xu *et al*, 2013) endocytosis in the calyx of Held. Along the same lines, acute cleavage of SNAP-25 and syntaxin 1 also reduced both fast and slow endocytosis in the calyx of Held (Xu *et al*, 2013). In addition, knocking down SNAP-25 or synaptobrevin resulted in slowing down clathrin-dependent endocytosis in cultured neurons (Zhang *et al*, 2013), although such synapses can nevertheless still endocytose to some extent (Neale *et al*, 1999).

Two interpretations appear possible. First, the endocytotic machinery needs to physically remove the SV SNARE molecules from the active zone. After SNARE cleavage the remaining membrane-bound fragments may be less well recognized by the endocytotic machinery, which results in their persistence in the active zone. Alternatively, SNARE cleavage or removal by knock-down approaches may result in a lower buffering of endocytotic cofactors in the synapse. The cofactors that are kept within synapses by binding to SNAREs will be partially lost, which will result in a lower endocytotic capacity, and slower endocytotic kinetics. In this context it is important to note that the control over the endocytosis process seems to be influenced by cell-wide factors, such as general protein availability, rather than by local factors (Armbruster & Ryan, 2011).

The physical link from exo- to endocytosis is still debatable. Much thought has been given, for example, to how fused vesicle patches move to the peri-active zone area to be endocytosed. However, it is unclear whether the peri-active zone is indeed a special area for endocytosis; for example, electron microscopy observations have given evidence of endocytosis at various sites, not only in the near-active zone area (Miller & Heuser, 1984; Rizzoli & Betz, 2004). An interesting connection, based on individual protein-protein interactions, has been made between active zone proteins (including piccolo, bassoon, ELKS/*Bruchpilot* and RIM) and endocytotic proteins such as dynamin, intersectin, AP2 and stonin (see for example Haucke *et al*, 2011 and references therein). Since the latter are involved in SV cargo recognition (see step 16), it can be envisioned that this chain of interactions represents a physical link between the fusion of the vesicle and its eventual endocytosis. This, however, still needs to be fully demonstrated. The main problem with this type of interpretation is that exo- and endocytosis are not particularly well linked at the level of the single vesicle. The fusion of one set of SVs typically results in the endocytosis of another set (Wienisch & Klingauf, 2006; Opazo & Rizzoli, 2010; Opazo *et al*, 2010). Also, exocytosis does not necessarily trigger endocytosis at single synaptic boutons, and endocytosis could be triggered in the absence of preceding exocytosis (Gandhi & Stevens, 2003), which renders the exo- to endocytosis connection rather loose.

Interestingly, a remarkable link between exocytosis and endocytosis at a genetic level has been recently described by investigations of mouse dynamin knock-out neurons (Lou *et al*, 2012). These synapses contained fewer synaptic vesicles, due to defects in endocytosis, but responded by increased Ca^2+^/calmodulin-dependent protein kinase II (CaMKII) activity. This results in increased phosphorylation of synapsin (Ferguson *et al*, 2007; Raimondi *et al*, 2011), which unbinds from the vesicles and presumably enhances their mobility and their ability to reach active zones and thus maintain exocytosis (see step 27; Denker *et al*, 2011).

### 34 Kiss-and-run

Molecular mechanisms are not sufficiently understood to draw a table for this reaction.

A special case of SV fusion is kiss-and-run, in which the vesicle may fuse only transiently with the plasma membrane, after which the fusion pore closes (Fesce *et al*, 1994; Alabi & Tsien, 2013). The main problem with understanding kiss-and-run is that much of the evidence presented over the last decade in favor of this model has been subsequently argued against on technical grounds, leaving readers with little uncontested evidence. For example, the speed of endocytosis measured by capacitance recordings (Sun *et al*, 2002) or by fluorescence imaging (Gandhi & Stevens, 2003) was used as an argument for kiss-and-run. Both types of experiments have later been claimed to report artifacts (Yamashita *et al*, 2005; Granseth *et al*, 2006).

The most advanced experiments describing kiss-and-run have relied on pH-sensitive quantum dots. These highly stable fluorophores that are quenched by the acidic pH inside vesicles, fluoresce brightly upon fusion, when the vesicle lumen switches to the neutral pH of the extracellular buffer (Zhang *et al*, 2007). The rapid changes in fluorescence (which take far less than the seconds or tens of seconds necessary for clathrin-mediated endocytosis) suggests that a type of kiss-and-run fusion takes place in a significant proportion of exocytosis events (Zhang *et al*, 2009; Park *et al*, 2012). Some controversy still remains, however (Granseth *et al*, 2009).

From a molecular point of view, few proteins have been linked to kiss-and-run so far. It has been suggested that perturbing clathrin removes all vesicle recycling (Granseth *et al*, 2006; Heerssen *et al*, 2008). One candidate is endophilin. The endocytosis of small vesicles through mechanisms independent of clathrin and AP2 seems to involve endophilin, and takes place on time scales faster than those of clathrin-dependent endocytosis, thus resembling kiss-and-run mechanisms to some extent (Llobet *et al*, 2011) (but note that endophilin is also known to be involved in clathrin-mediated endocytosis; Milosevic *et al*, 2011). A low requirement for clathrin in synaptic vesicle endocytosis has been suggested in *C. elegans* (Sato *et al*, 2009), where an ultra-rapid, clathrin-independent synaptic endocytosis pathway has also been recently described (Watanabe *et al*, 2013b,). However, the latter does not seem to correspond to kiss-and-run, as it involves the uptake of flattened, large pieces of membrane.

### 35 SNARE sorting (Fig 2H)

**Table:** 

Reaction	SNARE complexes are separated into single molecules by the activity of NSF and α -SNAP (or possibly β -SNAP; Burgalossi *et al*, 2010).
Partners	SNARE complexes in the plasma membrane (virtually immobile in comparison with the soluble partners), NSF, α -SNAP (soluble).
The trigger	Formation of SNARE complexes after fusion?
The control mechanism	NSF and α -SNAP are maintained in the synapse by interactions with SNAREs present on both the plasma membrane and on the SVs from the SV cluster. The triple SNARE complexes are bound with higher affinity by these molecules than the single SNAREs, and therefore their formation triggers the recruitment of NSF and α -SNAP.

After fusion one of the most important sorting steps would be the separation of the entangled SNARE molecules, as a result of NSF activity (see step 14). Blocking NSF activity, as in the *Drosophila* comatose mutant (Kawasaki *et al*, 1998; Littleton *et al*, 2001), results in a relatively rapid inhibition of synaptic release. The inhibition of NSF activity does not result in a lack of SNAREs available for fusion, since only a few are needed (step 31), and many are available (˜70 per SV; Takamori *et al*, 2006). However, it probably results in a poor sorting of SV molecules at the active zone, which induces difficulties in clearing the active zones and in allowing subsequent fusion events (step 33).

It has been observed that SNARE clusters increase the probability that NSF is located in their vicinity (Bar-On *et al*, 2009). Thus, it is likely that the presence of numerous SNAREs both on the vesicles (Takamori *et al*, 2006) and in clusters in the plasma membrane (Sieber *et al*, 2007; Bar-On *et al*, 2012) would result in the local buffering of NSF/α-SNAP in the synapse. Also, as α-SNAP/NSF complexes probably interact with higher affinity with the SNARE complexes than with single SNAREs, the formation of SNARE complexes would recruit the cofactors and induce SNARE disassembly (see table for this step).

## Degradation of the vesicle

### 36 Proteasomal degradation of SV components (similar to Fig 1F)

**Table:** 

Reaction	Unfolded protein is targeted by proteasomal components.
Partners	Unfolded SV protein (attached to SV surface?), proteasomal system (soluble).
The trigger	Unfolding of SV protein (spontaneous).
The control mechanism	Interactions with SVs keep the proteasomal components around SVs, and make them continually probe SV surfaces. High-affinity interactions ensue upon unfolding of SV protein.

Some proteins may already be changed, unfolded or otherwise damaged after recycling and may get targeted for degradation, possibly by the ubiquitin proteasome system (UPS). Mono-ubiquitination acts as a regulatory element in membrane trafficking (Hicke, 2001), but adding more ubiquitin molecules (poly-ubiquitination) will target proteins for degradation. Both mono- and poly-ubiquitination may be involved in synapses, allowing the UPS to regulate synaptic activity by controlling the abundance of key synaptic components (Chen *et al*, 2003; Speese *et al*, 2003; Willeumier *et al*, 2006; Yao *et al*, 2007).

Overall, the degradation of synaptic proteins is poorly understood, although some of the enzymes involved have been discovered over the last decade (see, for example, reviews in Yi and Ehlers (2007) and Segref and Hoppe (2009)). More discoveries are to be expected since synaptic proteasome research has been gaining momentum during the last few years, especially due to potential links to neurodegeneration (for example, Sharma *et al*, 2011, 2011, 2012). The reaction is probably based on the high-affinity interaction of UPS components with unfolded proteins (see table for this step; note similarity to step 10).

### 37 Lysosomal targeting

Presumably similar to step 5.

Damaged vesicles may be targeted for degradation in lysosomes, a process which takes place outside of the synapse, in the cell body, and implies the need for trafficking to the cell body. Retrograde trafficking of such organelles has been observed in the past, along microtobules, paralleling anterograde trafficking (step 9; see Tsukita & Ishikawa, 1980; Li *et al*, 1992; Li & Dahlström, 1997). This is another poorly understood process. Note that these organelles are not identical to synaptic vesicles in terms of morphology. Perhaps they are derived from constitutive endocytosis at the plasma membrane, or from the fusion of damaged vesicles to other organelles such as endosomes.

The damaged organelles need to be recognized by a pathway able to deliver them to the lysosome, possibly by proteins targeting unfolded proteins or damaged protein complexes. A potential pathway is that governed by Rab7, a membrane-organizing protein that seems to be fairly abundant in synapses, although not as enriched as Rab3 or Rab5 (Pavlos *et al*, 2010). Rab7 interacts with dynein motors (Jordens *et al*, 2001; Johansson *et al*, 2007), leading and might be involved in retrograde trafficking (Saxena *et al*, 2005) (see also review Hirokawa *et al*, 2010). An interesting question is how Rab7 reaches the synapse. It cannot be translated within the synapse (since there are no presynaptic ribosomes), so it needs to be brought there as anterograde cargo. However, it will have a tendency to “hop onto” retrograde cargoes or dynein motors during transport. While we could come up with different hypotheses, it would be useful to investigate this process directly.

### 38 Fusion to the lysosome

Presumably similar to steps 2 or 5.

Upon arrival in the cell body the organelles can fuse to lysosomes, leading to the degradation of their components, especially as Rab7 seems to be involved in delivering such organelles to the lysosome (see, for example, a recent review Wang *et al*, 2011).

## Immobile vesicles: a buffer for cofactors

### 39 Long-term vesicle mobility

Reaction table similar to that for step 29.

Even if they do not get modified and degraded, it is unlikely that the newly recycled vesicles will remain mobile for too long. As indicated at step 27, the recycling vesicles are mobile, they can reach the active zone, and are then released and recycled, which keeps them in the mobile pool. However, it is to be expected that sooner or later they will become entangled with synapsin, and will become trapped on the edges of the vesicle cluster (Rizzoli & Betz, 2004).

As a compensatory mechanism, synapsin may let go of some of the vesicles that it traps. It has been observed over more than two decades that synapsin disperses from the vesicle clusters upon synaptic activity (Torri-Tarelli *et al*, 1990; Chi *et al*, 2001), probably due to its calcium-induced phosphorylation triggered by the calcium entry that follows neuronal activity (see reviews in Cesca *et al* (2010) and Hilfiker *et al* (1999)). The SVs that have lost their synapsin could participate in recycling. Finally, mechanical tension within axons may also contribute to vesicle clustering, through mechanisms which are still under investigation (Siechen *et al*, 2009).

### 40 The reserve pool

Not a reaction: explanatory step.

A few mobile vesicles slowly interchange with a larger immobile fraction. For obvious reasons, the mobile vesicles recycle much more often than the stable, immobile ones. The recycling vesicles were named “the recycling pool” in the past, while the others were termed “the reserve pool”. The few lucky recycling pool vesicles docked to the active zone formed the “readily released pool”, or RRP, i.e., the first vesicles that release upon the arrival of an action potential train.

I will not discuss this terminology or the data that led to it in detail, as the various arguments have already been presented (Rizzoli & Betz, 2005; Denker & Rizzoli, 2010) (see also Pan & Zucker, 2009 for a quantitative model of vesicle pool activity). The main point to state here is that the conceptual framework discussed above predicts that there are no major molecular differences between reserve and recycling vesicles. This is most likely to be the case. Modulation of CDK5 (also mentioned under step 18) has been proposed to change reserve into recycling vesicles, without apparent major changes to the vesicles themselves (Kim & Ryan, 2010). Synapsin coming off vesicles would do the same, liberating the vesicles and allowing them to reach the active zone and to fuse (step 39; see Denker *et al*, 2011). Note also that CDK5 phosphorylates synapsin (Matsubara *et al*, 1996), and may coexist with it in protein complexes (Rosales *et al*, 2000), suggesting that these two mechanisms are related, if at all different (see also Kim *et al*, 2009 for further molecules involved in modulating the balance between reserve and recycling vesicles). One additional difference between recycling and reserve vesicles would be that recycling vesicles may fuse more often to endosomes, and may thus receive a larger fraction of endosomal proteins such as the endosomal SNAREs (Hoopmann *et al*, 2010).

Nevertheless, I conclude that the same vesicles may in turn be a recycling or a reserve pool, depending on how mobile they are at a particular point in time. Differences in the activity regime of the synapse (with or without the influence of post-synaptic signaling; Kauwe & Isacoff, 2013), likely cause the recruitment of reserve vesicles into the recycling pool (Lee *et al*, 2012), or *vice versa*.

### 41 How many vesicles are actually recycled at any one point in time?

Not a reaction: explanatory step.

This limited molecular difference between the different pools of vesicles indicates that the reserve vesicles could all, in principle, fuse upon stimulation (although only a few are ready for release at any time point; Trigo *et al*, 2012). Many, indeed, are able to do so, in a variety of synaptic systems, if sustained stimulation is applied (Rizzoli & Betz, 2005; Denker & Rizzoli, 2010; Xue *et al*, 2013). However, they do not appear to do so under *in vivo* conditions. The amount of synaptic vesicle recycling that synapses undergo in living animals appears to be limited: only a small percentage of the vesicles undergo recycling at any one time, at least for large, neuromuscular junction synapses (Denker *et al*, 2011, 2011), but also in some CNS synapses (Körber *et al*, 2012; Marra *et al*, 2012).

*In vivo*-like stimulation triggers the release of substantially more vesicles in Schaffer collateral boutons from brain slice cultures *in vitro* (Rose *et al*, 2013), albeit only the smallest synapses appeared to release all of their vesicles. Larger ones tend to release fewer vesicles upon stimulation (about 20% for the largest synapses analyzed, containing ˜400–500 vesicles) (Rose *et al*, 2013). Sustained stimulation at physiological frequencies also caused the eventual fusion of all vesicles in the calyx of Held (Xue *et al*, 2013) or in autaptic cultures (Ikeda & Bekkers, 2009). These findings are not in disagreement with the recycling of a limited number of vesicles *at any one time point* in synapses subjected to physiological activity. It is entirely expected that repeated activity would eventually use all vesicles, since this has already been demonstrated by classical studies employing repeated stimulation (Ceccarelli *et al*, 1972, 1973; Heuser & Reese, 1973). But this type of activity did not use up all vesicles at one time, but rather slowly, especially when *in vivo*-like patterns were used (Ceccarelli *et al*, 1972, 1973). However, only a relatively short interval was necessary to release most vesicles using *in vivo*-like stimulation patterns, in the recent *in vitro* studies mentioned above (Ikeda & Bekkers, 2009; Rose *et al*, 2013; Xue *et al*, 2013). This is possibly due to the fact that in these studies mammalian cells were stimulated at room temperature, at least about 12–13°C below the normal temperature of the rodent (In & On, 1932). The low temperature reduces the endocytotic capacity of the neurons (Fernández-Alfonso & Ryan, 2004), and forces them to use more vesicles than at normal physiological temperatures.

A rich literature from the synaptic firing field is in agreement with the suggestion of limited vesicle recycling *in vivo* (see also Denker *et al*, 2011 for further discussion). Most synapses do not release many vesicles upon stimulation *in vitro*: for example, the *cutaneous pectoris* NMJ of the frog releases only a few hundred vesicles per action potential (Katz & Miledi, 1979); although it contains hundreds of thousands of vesicles, and it can recycle the fused ones within a minute or less (Rizzoli & Betz, 2005). *In vivo*, this NMJ is probably active only in short, infrequent bursts of action potentials, which would not use many of its vesicles (Banner & Herrera, 1986).

The synapses that have been best studied *in vivo* are those from mammalian muscle. For example, physically active muscle synapses (the ones that fire in bursts to prompt rapid movements) may fire as little as 0.04–0.22% of the time. The amount of activity in tonic synapses, which fire continuously during movement, increases to approximately 20–35% of the time (Hennig & Lømo, 1985) (see also Eken (1998) and Eken *et al* (2008) for the development of firing patterns in muscles *in vivo*). It is likely that the tonic synapses also have larger vesicle pools, helping them sustain these stronger release patterns (Connor *et al*, 1997; Reid *et al*, 1999; Bewick, 2003).

The probability for a vesicle to be released by a single impulse is rather low, only about 10% (Slater *et al*, 1992) (see also Lorteije *et al*, 2009 for a CNS synapse). Even when such impulses come in bursts of high frequency (up to about 100 Hz, the standard frequency for phasic synapses), a burst may only release about one vesicle per active zone (Slater, 2003), which is nevertheless still several-fold higher than what is required to generate action potentials in the muscle (Wood & Slater, 1997, 2001). Thus, substantial release is simply not necessary under normal movement patterns.

But what about situations when the muscles would be pushed to the limit? Would then the reserve vesicles be necessary? This was not the case in grasshoppers which were being actively chased by predators (Denker *et al*, 2011). In humans, this type of experiment has been performed by measuring fatigue in muscle transmission (Merton, 1954; Stephens & Taylor, 1972; Bigland-Ritchie *et al*, 1982, 1983; Bellemare *et al*, 1983; Bigland-Ritchie & Woods, 1984; Irintchev & Wernig, 1987). The overall conclusion is rather simple: the first to fatigue are not the synapses, but the muscles. In other words, the synapses can be pushed to release large vesicle amounts, but the muscles will cease to respond, so that the release of the reserve pools would make little sense. Additionally, CNS pathways adapt to muscle fatigue and reduce the activation of motor neurons *in vivo*, limiting NMJ vesicle release.

Finally, even physiological frequencies of nerve stimulation can be more than what the muscle can bear. Mice allowed to perform voluntary, uncoerced running (Irintchev & Wernig, 1987; Wernig *et al*, 1990; Dorlöchter *et al*, 1991) exhibited leg muscle damage, perhaps because boredom or psychological damage leads to noxious levels of activity in mice in captivity. Thus, any increase in NMJ activity is unlikely to be well received by the muscles. Overall, this supports that synaptic activity *in vivo* is not as intense as we could assume, therefore many of the vesicles remain immobile and non-recycling at any one time.

### 42 A buffer pool, not a reserve pool

Reaction table similar to step 18.

These immobile reserve vesicles are a precious commodity, not an encumbrance to the synapse. Although they do not recycle, they retain the ability to interact with all the soluble proteins that the recycling vesicles interact with, proteins involved in both exo- and endocytosis. Many such interactions have been discussed so far. They are all of relatively low affinity, suggesting that the proteins will bind, rapidly come unbound, and then bind again to the vesicles. The vesicles thus act as a buffer for soluble proteins, in the same fashion in which calcium-binding proteins act as calcium buffers (Denker *et al*, 2011). This is a simple and powerful means of maintaining a large assortment of soluble proteins in the synapse. This type of buffering has been suggested for multiple proteins, including several endocytosis cofactors (Denker *et al*, 2011), and has recently been demonstrated for complexin in molecular detail (Wragg *et al*, 2013).

This appears to be an important potential use for the immobile fraction of vesicles – although possibly not the only one. Others have been suggested in the past: for example, these vesicles may be a reservoir of neurotransmitter. However, the fact that they do not appear to exchange their neurotransmitter contents with other elements (reviews Ceccarelli & Hurlbut, 1980; Van der Kloot, 2003) suggests that this is not their main utility in the synapse. A second possibility is that they may engage in spontaneous fusion (see next step).

### 43 The complex issue of spontaneous vesicle fusion

Reaction table similar to that for step 31 (fusion). It may represent spontaneously forming A'B products.

That vesicles can fuse spontaneously (independent of calcium influx; Vyleta & Smith, 2011) has been noted for about six decades (Del Castillo & Katz, 1954). This may be due either to the spontaneous fusion of recycling vesicles, or to the activity of a separate class of vesicles that mostly fuse in spontaneous fashion, such as reserve vesicles. This is still an open question. Some simple experiments tend to suggest that all vesicles are similar: all vesicles can be stimulated to release and recycle (for example, Heerssen *et al*, 2008; Denker *et al*, 2011). Conversely, virtually all vesicles can release spontaneously after treatments that promote spontaneous activity (Henkel A & Betz, 1995; Rizzoli & Betz, 2002). Labeling spontaneously or actively releasing vesicles with fluorescent dyes has indicated that these populations are non-overlapping, partially-overlapping (Sara *et al*, 2005; Mathew *et al*, 2008; Fredj & Burrone, 2009; Chung *et al*, 2010), or fully overlapping (Groemer & Klingauf, 2007; Hua *et al*, 2010; Wilhelm *et al*, 2010). Different treatments may promote either active or spontaneous release (for example, Nosyreva & Kavalali, 2010; Sara *et al*, 2011), although it is unclear whether this indicates that a different pool of vesicles is activated in each case.

The overexpression of certain endosomal SNARE proteins seems to place these molecules in a pool of vesicles that preferentially recycle in the absence of stimulation (Hua *et al*, 2011b; Raingo *et al*, 2012; Ramirez *et al*, 2012), or, on the contrary, in a pool of vesicles that recycle upon stimulation (Hoopmann *et al*, 2010). One interpretation of this type of investigations is that it is extremely difficult to differentiate the spontaneous fusion of “true” vesicles from the spontaneous fusion of their precursors (step 13). Additionally, upon overexpressing minor components of synaptic vesicles, such as the endosomal SNAREs (Takamori *et al*, 2006), the molecules that do not fit into the vesicles may be shunted to a constitutively fusing population of precursors or endosomal vesicles, which may explain observations of spontaneous fusion for these molecules. After all, even synaptobrevin, a *bona fide* vesicle component, is partially mislocalized upon overexpression (Pennuto *et al*, 2003). Thus, it is likely that the vesicles from the recycling pool, which fuse upon stimulation, also occasionally fuse in spontaneous fashion, perhaps relying for this on specific proteins (Burgalossi *et al*, 2010) such as Doc2 (Groffen *et al*, 2010; Pang *et al*, 2011). Note also that Doc2 has also been implicated as a sensor for asynchronous neurotransmitter release (Yao *et al*, 2011).

Finally, it is likely that spontaneous release represents an important biological phenotype, involved, for example, in maintaining synapses. Presynaptic boutons that do not release neurotransmitter are disengaged from the postsynaptic boutons (see, for example, Richards *et al*, 2005). It is possible that spontaneous release is necessary to prevent this from happening to synapses that are silent for long periods. Alternatively, many of the conflicting results observed by different laboratories may be due to investigating different developmental stages of the neurons. In cultured neurons the spontaneous release is much stronger in young neurons than in mature ones (Andreae *et al*, 2012), which will thus induce different results in cultures of different ages.

## Pushing the limits of recycling

### 44 Excessive synaptic release results in too much material on the plasma membrane

This is a simple consequence of limited resources (principle iv). The mechanisms are still too poorly understood to allow the description of a complete reaction.

As indicated in step 41, not many vesicles recycle at any one time. When stimulation surpasses the ability of the synapse to recycle vesicles (as often observed *in vitro*), all vesicle pools are depleted and are only slowly regenerated. The vesicle membrane added into the plasma membrane either swells the nerve terminals or folds back onto itself, generating what has been termed “infoldings”. This process, also known as “bulk endocytosis,” is probably an emergency mechanism, with limited physiological significance for synapses that recycle only few vesicles at a time, such as the *cutaneous pectoris* frog NMJ (step 41; see also Richards *et al*, 2003), and which may be used by other synapses only during rare high-activity bursts (Gaffield *et al*, 2009). It may be physiologically relevant for synapses that fire prolonged high frequency bursts under normal, physiological conditions (see Rizzoli & Betz, 2005; Clayton *et al*, 2007; Cheung *et al*, 2010). In such synapses bulk endocytosis may be necessary to remove the newly added membrane from the plasma membrane, to in order to prevent damage to the synapse. Different endocytotic molecules may be involved, versus normal clathrin-mediated endocytosis, including the glycogen synthase kinase 3 (GSK3) (Clayton *et al*, 2010).

### 45 Why bulk endocytosis is necessary

Same as 44.

But why is clathrin-mediated endocytosis unable to deal with the flow of vesicles in these cases? The simplest hypothesis is that the endocytosis machinery contains too few cofactor molecules to retrieve an indefinite number of SVs. The early observations of Heuser and Reese (1973) demonstrated that strong stimulation (1 minute at 10 Hz) causes the depletion of tens of thousands of vesicles, and the formation of only a few hundred coated vesicles in the frog NMJ – in fact, about 1 for each 30 depleted synaptic vesicles. All vesicles were eventually retrieved, over many minutes, suggesting that the clathrin machinery processed the membrane very slowly, retrieving a few vesicles at a time, then disengaging the coat (uncoating) and proceeding to the next set of vesicles. This was underlined in later experiments in which the simultaneous release of at least approximately 10–20 vesicles per active zone was followed by the rapid clathrin-mediated recycling of only 5–6 of them, with the rest waiting for the clathrin machinery to become available (Miller & Heuser, 1984) (see also Heuser *et al* (1979), Heuser and Reese (1981) and discussion in Denker *et al* (2011)).

As indicated under step 42, clathrin machinery components are probably kept within the synapse by being buffered by the “reserve” SVs. It is highly likely that each of these SVs only interacts with a few components of this machinery at a time, which indicates that a relatively low number of components can be kept within the synapse at any one time. This limits the amount of endocytosis that can be performed, and thus imposes an upper limit on synaptic activity.

### 46 Bulk endocytosis mechanisms

Same as 44.

The molecular mechanisms of bulk endocytosis are still disputed, although it is fairly certain that, at least in some synapses, all endocytosis, including bulk, requires GTP-ases, and probably dynamin as well (step 19; Jockusch *et al*, 2005). The presence of dynamin on membranes that appear to be in the process of bulk endocytosis further suggests the involvement of this molecule (Takei *et al*, 1996). Additionally, membrane formations resembling bulk endocytosis tend to accumulate in dynamin-lacking synapses (Ferguson *et al*, 2007; Hayashi *et al*, 2008).

### 47 The clathrin machinery in resting synapses

Incomplete reaction: a simple consequence of limited resources (principle iv). Otherwise, the same as steps 4, 16 and 17.

One important point regarding the availability of clathrin and other endocytosis cofactors (step 45) is that they would probably follow their natural binding preferences at rest as well as during activity: a fraction of the cofactors would be buffered by the synaptic vesicles (step 41), but others would tend to bind to fused SV molecules on the plasma membrane. These assemblies of molecules may go as far as to form fully coated vesicles, although in this case they would probably complete endocytosis, and not remain on the plasma membrane. More likely, the amount of cofactor molecules available at rest is to some extent limiting, as most molecules are probably buffered by the vesicle cluster. Consequently the fused vesicles that have not yet been endocytosed (in the few seconds or tens of seconds after activity) would only partially be covered with endocytosis cofactors. As discussed under step 18, the entry of calcium during stimulation would tend to liberate cofactors from the vesicle cluster, allowing the completion of endocytosis events (Wienisch & Klingauf, 2006) (see also discussion in Rizzoli and Jahn (2007)).

## Long-term synaptic changes

### 48 Sharing vesicles between synapses

Reaction table similar to that for step 12.

An important element in the synaptic vesicle cycle is that vesicle proteins may interact occasionally with the motor proteins that brought them to the synapse in the first place. This would lead to inter-bouton movements tending to share material between neighboring synapses (Darcy *et al*, 2006; Westphal *et al*, 2008; Staras *et al*, 2010). The soluble molecules associated with vesicles would move as well, just as when they were transported to the synapse (step 7). This process homogenizes neighboring synapses, and, as a result, all the boutons along an axonal branch receive sufficient amounts of the newly formed material transported from the cell body. This material may otherwise tend to accumulate at microtubule ends, in the last boutons along one axon (step 12).

### 49 Changes in the number of vesicles within single synapses over time

Complex phenomenon, not a single reaction.

The balance of biogenesis and degradation renders vesicle populations relatively constant in synapses (Minerbi *et al*, 2009), unless some elements perturb the balance and require changes. Synapses silenced by the application of action-potential-inhibiting drugs grow larger, seemingly as a response to release limitations (for example, Murthy *et al*, 2001). A simple explanation is that the drugs limit synaptic vesicle recycling and protein activity, and therefore reduce protein damage. This would reduce the loss of damaged material to retrograde trafficking, while maintaining anterograde trafficking unchanged, and would result in increased synapse size. Normal levels of protein degradation are known to reduce synaptic activity, with proteasomal inhibition counteracting this effect (Jiang *et al*, 2010).

This argument cannot be used for changes such as the stimulation-induced, long-term potentiation of release observed in rodent brain slices (for example, Zakharenko *et al*, 2001). Here, more complex mechanisms, involving long-term calcium-induced changes to the recycling machinery need to be invoked. These mechanisms are only speculative at the moment, and may also not be very strictly linked to synaptic activity (Fisher-Lavie *et al*, 2011).

### 50 Synapse stability

Complex phenomenon, not a single reaction.

Nevertheless, the synapse is a remarkably stable and resistant sub-cellular structure. Synapses can still release vesicles upon stimulation after being broken from the axons in fairly damaging procedures (Nicholls & Sihra, 1986); and some neuromuscular synapses of amphibians can be stimulated to release and recycle vesicles for days *in vitro* (Ceccarelli *et al*, 1972, 1973; Rizzoli & Betz, 2002). This suggests that simple and effective mechanisms, such as those indicated above, control the recycling process at least in the short and medium term (hours to days), without much involvement of genetically-controlled mechanisms originating in the cell body.

## Conclusion

SV recycling is a complex process, but it is controlled by relatively simple principles. Each step in the vesicle cycle depends on the existence of a core structure of the vesicle, which may be as simple as an assembly of synaptophysin and cholesterol molecules. This recruits several other proteins that have binding partners within the synaptophysin/cholesterol environment, including, when available, synaptobrevin or synaptotagmin. This core vesicle structure acts as a buffer to recruit soluble cofactors. These determine the next step in the vesicle cycle, depending on location, on the composition of the membrane (Golgi, plasma membrane, complete SV, etc.), on cofactor availability, and on which cofactors are recruited by non-vesicular elements present in the same environment (such as PIP2 when the vesicle molecules are in the plasma membrane). The vesicle cluster in the synapse continues to behave in this way, also acting as a buffer for its binding partners and keeping them within the synaptic space.
